# Circadian gene BMAL1 ameliorates renal ischaemia-reperfusion injury in diabetic mice by enhancing mitophagy via the HIF-1/BNIP3 pathway

**DOI:** 10.1038/s41598-025-03515-5

**Published:** 2025-07-02

**Authors:** Xinqi Deng, Yan Leng, Yonghong Xiong, Wenyuan Li, Wu Chen, Yuhang Yang, Bihan Wang, Siyuan Gong, Yunhao Wang, Baichuan Yang, Wei Li

**Affiliations:** 1 Department of Anesthesiology, Renmin Hospital of Wuhan University, Wuhan, 430060 Hubei China; 2 Department of Urology, Renmin Hospital of Wuhan University, Wuhan, China

**Keywords:** BMAL1, Diabetes, HIF-1α, Ischemia/reperfusion injury, Mitophagy, Nephrology, Cell biology, Cell death

## Abstract

Diabetic kidneys are particularly vulnerable to ischemia/reperfusion injury (I/RI). Although previous research has suggested that the circadian gene brain and muscle ARNT-like 1 (BMAL1) plays a role in regulating renal function, the exact functions and mechanisms of BMAL1 in diabetic renal I/RI remain elusive. In this study, bilateral renal artery ligation and release were performed in non-diabetic (db/+) and diabetic (db/db) mice. In diabetic kidneys, experimental findings demonstrated a significant decrease in BMAL1 expression, along with the inhibition of the HIF-1α/BNIP3 signaling pathway and compromised mitophagy. BMAL1 overexpression alleviated cell damage and apoptosis under high glucose and hypoxia/reoxygenation stimulation. Inhibition of the Hypoxia-inducible factor-1α (HIF-1α)/ B-cell lymphoma-2 interacting protein 3 (BNIP3) pathway by the HIF-1α inhibitor PX-478 intensified cellular damage and reduced the protective effect of BMAL1 overexpression in TCMK-1 cells. These results indicate that BMAL1 regulates mitophagy in diabetic renal I/RI through the HIF-1α/BNIP3 pathway, providing valuable insights for the development of targeted therapies for diabetic renal I/RI.

**Supplementary Information:**

The online version contains supplementary material available at 10.1038/s41598-025-03515-5.

## Introduction

The prevalence of diabetes is steadily increasing worldwide and has received widespread attention due to the serious harm it brings. Mounting evidence indicates that diabetes not only directly induces kidney damage but also aggravates renal I/RI^1-3^. I/RI is a common contributor to acute kidney injury (AKI) in clinical practice, particularly during renal procedures such as partial nephrectomy and kidney transplantation^4^. Although many studies have been conducted on diabetic renal I/RI, the precise mechanism underlying diabetes leads to heightened susceptibility to renal I/RI remains unclear.

The circadian system is regulated by a central clock within the suprachiasmatic nucleus (SCN) of the hypothalamus, in coordination with peripheral oscillators dispersed throughout various tissues^5^. The circadian clock functions through an intricate system of interconnected transcription-translation feedback loops, where BMAL1 acts as a key regulator of clock genes. BMAL1 is crucial not only for orchestrating circadian rhythms but also for preserving normal renal physiological function^6,7^. Disruptions in the circadian regulation of clock genes in diabetic patients contribute to various pathophysiological processes^8^, with BMAL1 downregulation emerging as a key factor in the initiation and progression of diabetic nephropathy^9^. Studies have found that the circadian rhythm expression of clock genes is disrupted in the kidneys of db/db mice^10^. Additionally, mice with BMAL1 knockout exhibit disrupted sleep-wake rhythms and β-cell secretion disorders, which lead to hyperglycemia, impaired glucose tolerance, and a metabolic syndrome involving glucose and fatty acids, ultimately progressing to diabetes^11^. Recent studies highlight the critical impact of disrupted mitochondrial homeostasis on the aggravation of renal I/RI^12^. In particular, the downregulation of BMAL1 has been strongly linked to this adverse outcome^13-15^. BMAL1 was reported to mitigate ischemia/reperfusion (I/R) induced tubular damage and protect kidney function by directly upregulating the expression of antioxidant proteins and maintaining mitochondrial homeostasis^14,16^. Despite extensive investigation, the exact contribution of BMAL1 to diabetic renal I/RI remains poorly understood.

Mitophagy, a crucial component in maintaining mitochondrial homeostasis, helps alleviate renal I/RI by specifically identifying and timely removing damaged mitochondria^17^. However, mitophagy is suppressed in a diabetic kidney ischemia-reperfusion, which leads to more severe renal I/RI in diabetic rats^18^. HIF-1α is a protein containing the basic helix-loop-helix/Per-ARNT-SIM (bHLH-PAS) domain, recognized as the hallmark protein of cellular and tissue hypoxia and its levels markedly increase after renal I/RI^19^. In hypoxic environments, HIF-1α binds to the promoter of BNIP3, thereby enhancing its transcription^20^. BNIP3, a key mitochondrial outer membrane protein, plays a crucial role in mitophagy. Within renal tubular cells, the HIF-1α/BNIP3-mediated mitophagy alleviates the adverse impacts of I/RI by curtailing apoptosis and diminishing oxidative stress^21^. Research has revealed diminished serum levels of HIF-1α and BMAL1 in individuals with type 2 diabetes^22^, and the transcriptional activity of HIF-1α is known to be associated with BMAL1^23^. Additionally, a lack of BMAL1 in skeletal muscle correlates with reduced HIF-1α levels^24^. Despite these observations, the relationship between BMAL1 and HIF-1α/BNIP3-mediated mitophagy, particularly in the context of diabetic renal tubular cells, remains poorly understood. In this study, we conducted both in vivo and in vitro investigations to elucidate whether BMAL1 plays a protective role by promoting HIF-1α/BNIP3-mediated mitophagy in the context of diabetic renal I/RI. Our findings suggest a novel therapeutic avenue for targeted treatment of diabetic renal I/RI.

## Results

### General characteristics of the experimental animals before renal I/R injury

Table 1 highlights that db/db mice exhibited markedly elevated blood glucose levels and increased body weight compared to db/+ mice. These db/db mice displayed classic features of type 2 diabetes, including polydipsia, polyphagia, and polyuria.

**Table 1:** General characteristics of the experimental animals before renal I/R injury.

Parameter	db/+ mice	db/db mice
Blood glucose, mmol/L	5.95 ± 0.34	13.25 ± 0.93^*^
Body weight, g	21.42 ± 0.94	41.45 ± 1.32^*^

Results are expressed as means ± SD; *n* = 6. ^*^ *P* < 0.05 versus the db/+ mice.

### Renal I/R induces more severe renal injury and apoptosis in Db/db mice

**Fig. 1:**
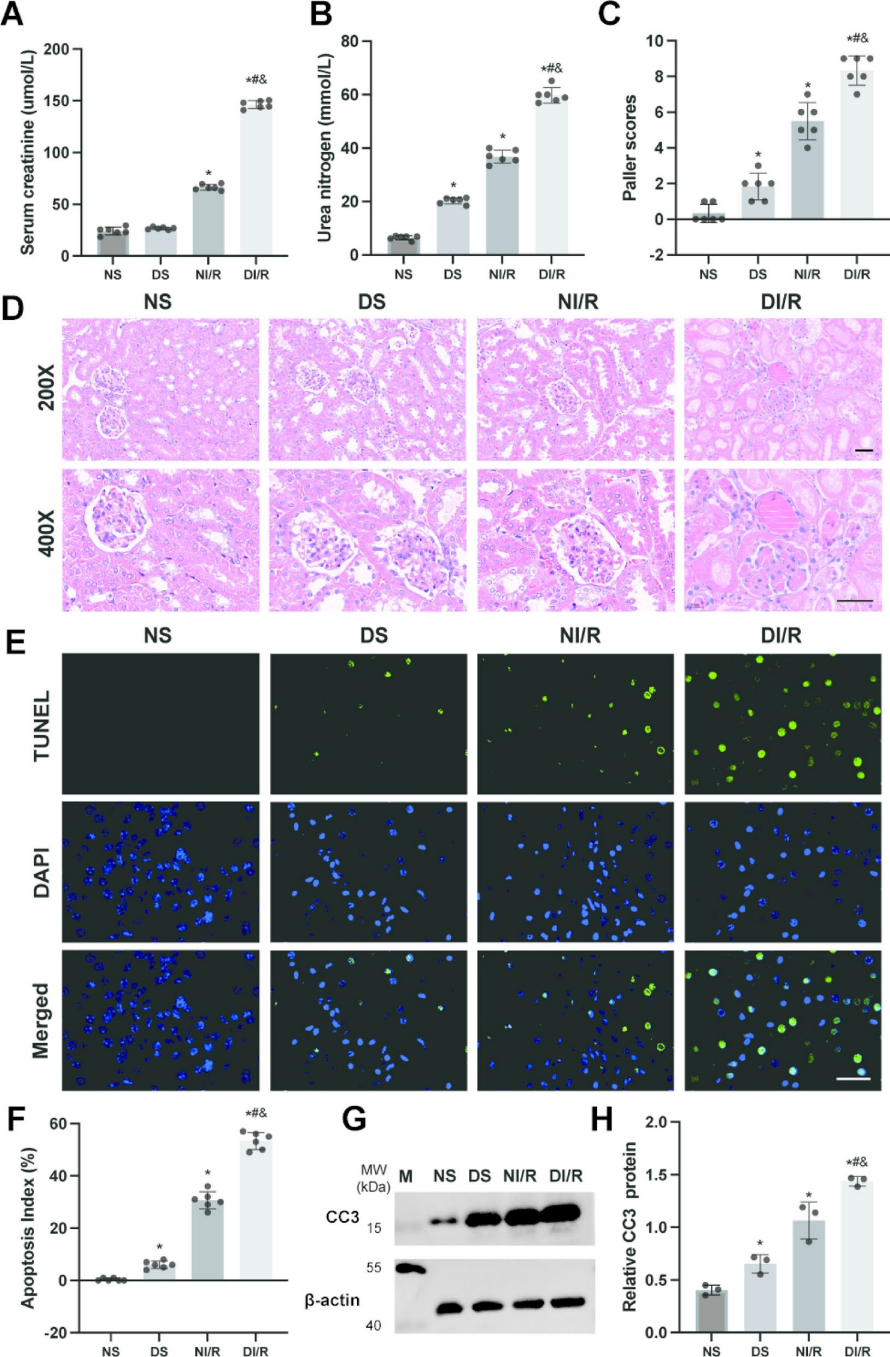
Renal I/R induces more severe renal injury and apoptosis in db/db mice. (**A**,** B**) Scr and BUN levels were measured from blood samples collected from each experimental group of mice, *n* = 6 independent experiments. (**C**) Renal tubular injury was assessed using the Paller score for each group, *n* = 6 independent experiments. (**D**) HE staining of the renal cortex was performed to evaluate renal tissue damage. (**E**) Cell apoptosis was detected using the TUNEL assay. (**F**) Quantification of TUNEL-positive cells was conducted. (**G**,** H**) Western blot analysis was employed to determine the expression levels of CC3 protein in renal tissue. Scale bar = 50 μm. Results are expressed as means ± SD; *n* = 3 independent experiments, otherwise specified. Statistical significance was indicated as *P < 0.05 compared to the NS group; #*P* < 0.05 compared to the DS group; &*P* < 0.05 compared to the NI/R group. Supplementary Fig. S1 includes both cropped images and full-length blots.

As illustrated in Fig. 1, mice in the DS group exhibited elevated serum creatinine (Scr) levels compared to the NS group, alongside an increase in blood urea nitrogen (BUN) levels. Similarly, the NI/R group demonstrated higher Scr and BUN levels compared to the NS group, indicating more pronounced renal dysfunction. Notably, following renal I/R, the DI/R group showed a substantial rise in both Scr and BUN levels relative to the NI/R group. These results underscore that renal I/R induces renal functional impairment, with diabetes further aggravating the damage caused by I/R.

In Fig. 1D, hematoxylin and eosin (HE) staining of renal tissue revealed that in the NS group of mice, the renal tubular structure appeared intact, exhibiting regular morphology without obvious swelling, expansion, degeneration, or necrosis. Conversely, in the DS group, there was a small amount of tubular dilation, hyaline degeneration and necrosis. The I/R group exhibited significant renal tubular epithelial cell damage, evidenced by vacuolar degeneration, cell shedding, tubular dilation, interstitial edema, cast formation, and infiltration of inflammatory cells. These pathological alterations were markedly more severe in the DI/R group compared to the NI/R group. Furthermore, Paller score analysis of renal tubular injury revealed that the I/R group had a substantially higher injury score than the sham surgery group, with the DI/R group demonstrating elevated scores relative to the NI/R group.

To evaluate apoptosis levels in kidney tissues across different mouse groups, we utilized TdT-mediated dUTP Nick-End Labeling (TUNEL) staining. This analysis revealed a significant increase in TUNEL-positive cells in the kidneys of the I/R group compared to the sham operation group. Similarly, the DI/R group displayed a notable rise in TUNEL-positive cells relative to the NI/R group (Fig. 1E, F). Additionally, Western blot analysis demonstrated elevated expression of cleaved caspase-3 (CC3) in both the NI/R and DI/R groups (Fig. 1G, H). Collectively, these findings suggest that diabetes exacerbates kidney damage induced by I/R.

### Diabetes reduces the expression of BMAL1 and the level of mitophagy and aggravates renal I/R-induced mitochondrial damage

**Fig. 2:**
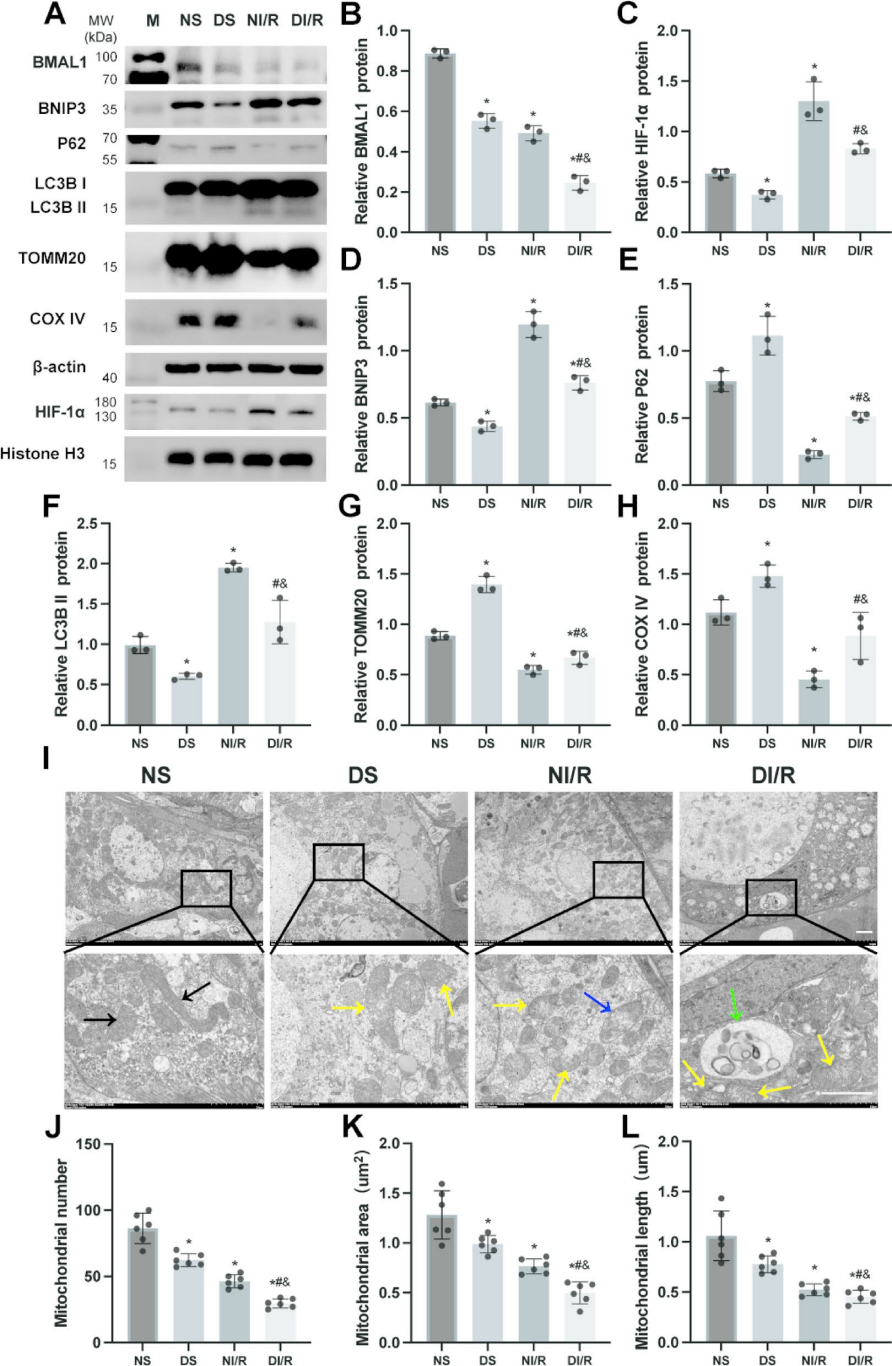
Diabetes reduces the expression of BMAL1 and the level of mitophagy and aggravates renal I/R-induced mitochondrial damage. Kidney cortex tissues were analyzed via Western blot to assess the expression levels of BMAL1, HIF-1α, BNIP3, p62, LC3B, TOMM20 and COX IV. (**A**) Representative Western blot images are shown; (**B**-**H**) Statistical analysis of BMAL1, HIF-1α, BNIP3, p62, LC3B II, TOMM20, and COX IV protein levels are presented. (**I**) Black arrows denote intact mitochondria, yellow arrows indicate damaged mitochondria, blue arrows point to mitochondrial autophagosomes, and green arrows highlight autophagosomes. (**J**) Average mitochondria number, *n* = 6 independent experiments. (**K**) Average mitochondria area analysis, *n* = 6 independent experiments. (**L**) Average mitochondrial lenght analysis, *n* = 6 independent experiments. Scale bar = 2 μm. Results are expressed as means ± SD; *n* = 3 independent experiments, otherwise specified. *P < 0.05 vs. the NS group; #*P* < 0.05 vs. the DS group; &*P* < 0.05 vs. the NI/R group. Supplementary Fig. S2 includes both cropped images and full-length blots.

To elucidate the alterations in BMAL1 expression and mitophagy during diabetic kidney I/R, we assessed the levels of BMAL1 and mitophagy-related proteins in the kidneys of mice across different experimental groups (Fig. 2A). Western blot analysis demonstrated a notable reduction in BMAL1 expression in the kidney I/R group compared to the sham operation group, indicating a disruption in the core clock genes’ activity (Fig. 2B). Concurrently, there was a marked increase in microtubule-associated protein 1 light chain 3B (LC3B) II levels and a decrease in p62, reflecting enhanced autophagy (Fig. 2E, F). Additionally, we observed a significant decline in mitochondrial membrane proteins translocase of outer mitochondrial membrane 20 (TOMM20) and cytochrome c oxidase IV (COX IV), suggesting increased mitochondrial degradation (Fig. 2G, H). Furthermore, there was a substantial upregulation of HIF-1α and BNIP3, pointing to an elevated mitophagy process mediated by the HIF-1α/BNIP3 pathway in the kidneys following I/R (Fig. 2C, D).

Compared to the non-diabetic cohort, the diabetic group exhibited reduced expression levels of BMAL1, HIF-1α, BNIP3, and LC3B II, while p62, TOMM20, and COX IV levels were significantly elevated. These findings suggest that renal I/R induces a downregulation of BMAL1 and activates the HIF-1α/BNIP3-mediated mitophagy pathway. Furthermore, diabetes exacerbates the suppression of BMAL1 expression, leading to a concomitant decline in mitophagy. Therefore, we speculate that renal I/RI responsive activates mitophagy, while in a high/glucose environment, BMAL1 expression is additionally suppressed, and endogenous protective mechanisms are deactivated, leading to a decrease in mitophagy levels and further aggravation of renal damage.

To evaluate mitochondrial morphology and mitophagy activation, we employed transmission electron microscopy to examine kidney samples from each experimental group, and assessed mitochondrial number, area, and length (Fig. 2I-L). As illustrated in Fig. 2I-L, our results confirm the Western blot findings, revealing a significant increase in the number of autophagosomes and mitophagosomes in the kidneys of mice subjected to I/R, while the number of mitochondria decreased, indicating an enhanced mitophagic response to renal ischemia-reperfusion. Furthermore, compared to the NS group, the DS group exhibited marked lipid droplet accumulation in renal tubular epithelial cells, with a reduction in the number, area, and length of mitochondria. In the I/R group, mitochondrial integrity was significantly compromised, with a decrease in mitochondrial area and length, and rupture, swelling, and fragmentation of the cristae were observed. Additionally, the DI/R group showed a significant increase in the number of damaged mitochondria, further exacerbating mitochondrial injury.

### HG aggravates TCMK-1 cell injury and mitochondrial dysfunction induced by H/R

**Fig. 3:**
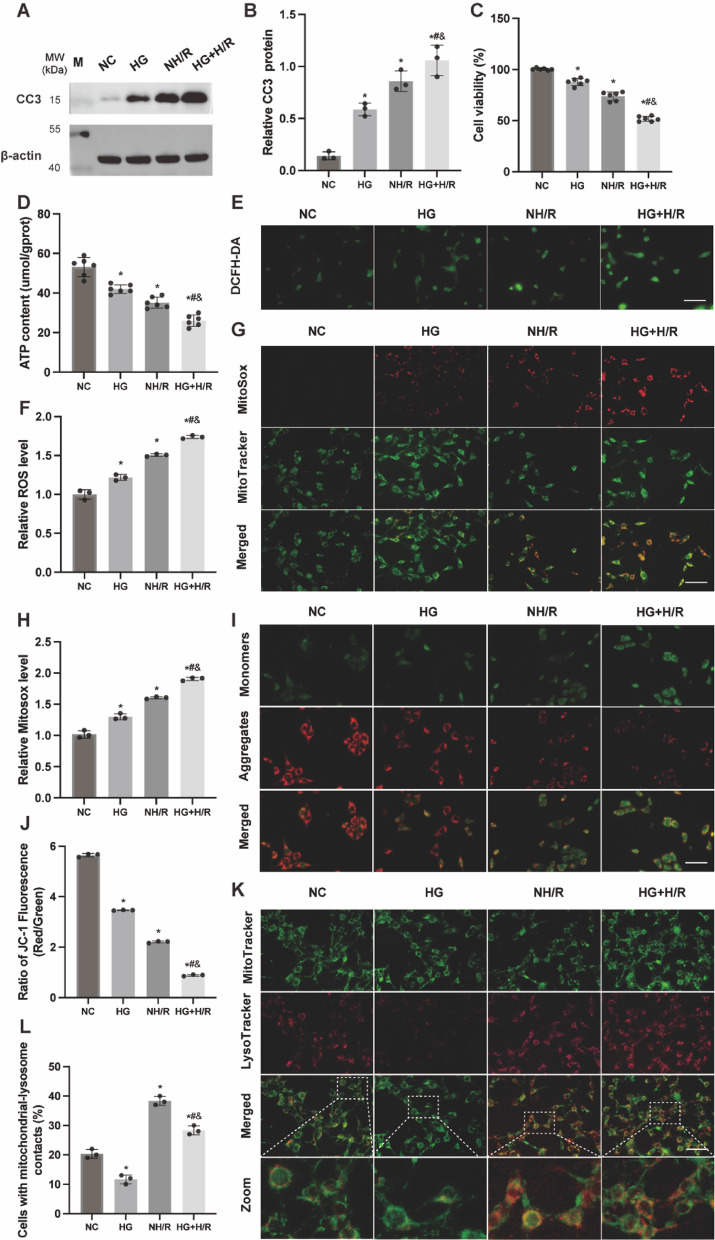
HG aggravates TCMK-1 cell injury and mitochondrial dysfunction induced by H/R. (**A**,** B**) WB analysis of CC3 protein expression levels in kidney tissue. (**C**) Cell viability detected by CCK8 assay, *n* = 6 independent experiments. (**D**) ATP content, *n* = 6 independent experiments. (**E**,** F**) ROS levels detected by DCFH-DA probe. (**G**,** H**) Mitochondrial ROS levels detected by MitoSox Red. (**I**) JC-1 staining observation of mitochondrial membrane potential. (**J**) JC-1 staining analysis of the ratio of green/red fluorescence intensity, reflecting MMP levels. (**K**) Representative fluorescent images of lysophagy in each group. (**L**) Quantification of K. Scale bar = 50 μm. Results are expressed as means ± SD; *n* = 3 independent experiments, otherwise specified. *P < 0.05 vs. the NC group; #*P* < 0.05 vs. the HG group; &*P* < 0.05 vs. the NH/R group. Supplementary Fig. S3 includes both cropped images and full-length blots.

Cell viability was evaluated using the Cell Counting Kit-8 (CCK-8) assay, while Western blotting was employed to quantify CC3 as a marker of apoptosis across experimental groups. Our results demonstrate that both HG and H/R conditions lead to cellular damage, decreased viability, and increased apoptosis. Particularly, H/R alone caused more severe cellular injury, with the HG + H/R group exhibiting the most significant damage (Fig. 3A, B, C). Mitochondrial function was assessed by measuring adenosine triphosphate (ATP) levels, reactive oxygen species (ROS) production, mitochondrial ROS and mitochondrial membrane potential (MMP) (Fig. 3D-J). Intracellular ROS and mitochondrial ROS levels were determined by the intensity of fluorescence from dichlorofluorescein (DCF) and MitoSox Red, with increased fluorescence indicating higher ROS levels. MMP alterations were visualized using JC-1 staining: red fluorescence denoted healthy mitochondria, while green fluorescence indicated a loss of MMP, with an increased green/red fluorescence ratio reflecting mitochondrial damage. Our findings reveal that both HG and H/R conditions result in diminished ATP levels and MMP, alongside elevated ROS levels. Notably, the HG + H/R condition resulted in the most pronounced decline in ATP and MMP, and the highest ROS levels. These results suggest that HG exacerbates H/R-induced mitochondrial dysfunction and damage in TCMK-1 cells, corroborating previous animal model studies. Additionally, we performed fluorescence colocalization analysis using Mito-Tracker Green and Lyso-Tracker Red to visualize mitochondria and lysosomes, respectively. The results showed an increase in mitochondria-lysosome fusion events in the hypoxia-reoxygenation group, while a decrease was observed in the high-glucose group, suggesting that mitophagy is inhibited under high-glucose conditions.

### The expression of BMAL1 and the level of mitophagy is reduced in TCMK-1 cells exposed to HG

**Fig. 4:**
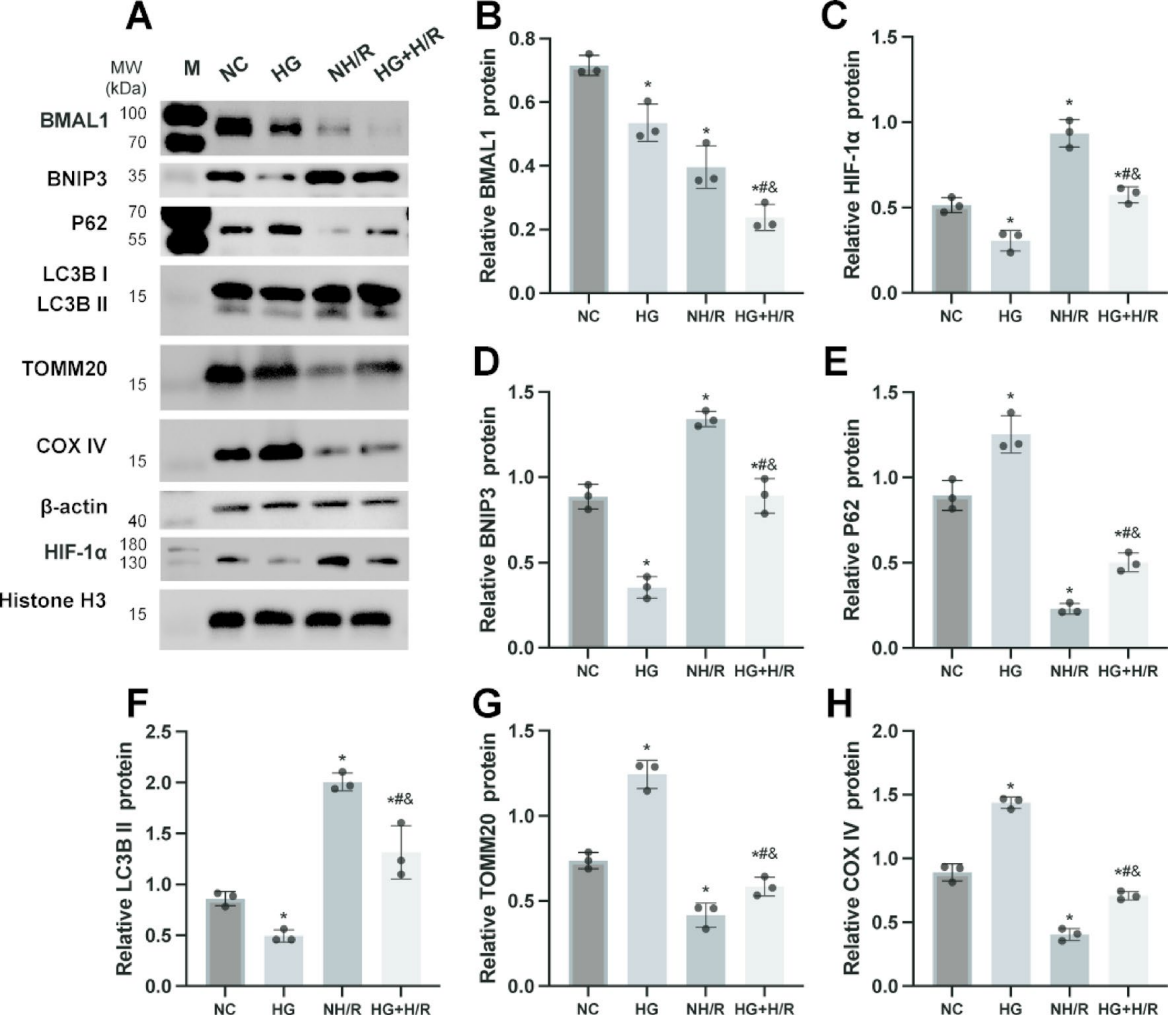
The expression of BMAL1 and the level of mitophagy is reduced in TCMK-1 cells exposed to HG. Cells from each group were collected, and WB was used to detect the expression of BMAL1, HIF-1α, BNIP3, p62, LC3B I, LC3B II, TOMM20, and COX IV proteins. (**A**) WB image. (**B**-**H**) Statistical analysis of BMAL1, HIF-1α, BNIP3, p62, LC3B II, TOMM20, and COX IV expression. Results are expressed as means ± SD; *n* = 3 independent experiments, otherwise specified.^*^P < 0.05 vs. the NC group; ^#^*P* < 0.05 vs. the HG group; ^&^*P* < 0.05 vs. the NH/R group. Supplementary Fig. S4 includes both cropped images and full-length blots.

To investigate the effects of HG and H/R on cellular processes, we employed an in vitro model using TCMK-1 cells. Western blot analysis (Fig. 4) demonstrated that in cells subjected to NH/R, there was a significant increase in the expression levels of HIF-1α, BNIP3, and LC3B II compared to the NC group, while BMAL1, p62, TOMM20, and COX IV levels were notably decreased. This suggests that H/R induces a downregulation of BMAL1 and activates mitophagy in TCMK-1 cells. Conversely, in cells exposed to HG + H/R, the levels of BMAL1, HIF-1α, BNIP3, and LC3B II were diminished relative to the NH/R group, whereas p62, TOMM20, and COX IV levels were elevated. These results indicate that high glucose further inhibits BMAL1 expression and diminishes mitophagy, corroborating our animal experiment findings.

### Overexpression of BMAL1 induces mitophagy mediated by the HIF-1α/BNIP3 pathway

**Fig. 5:**
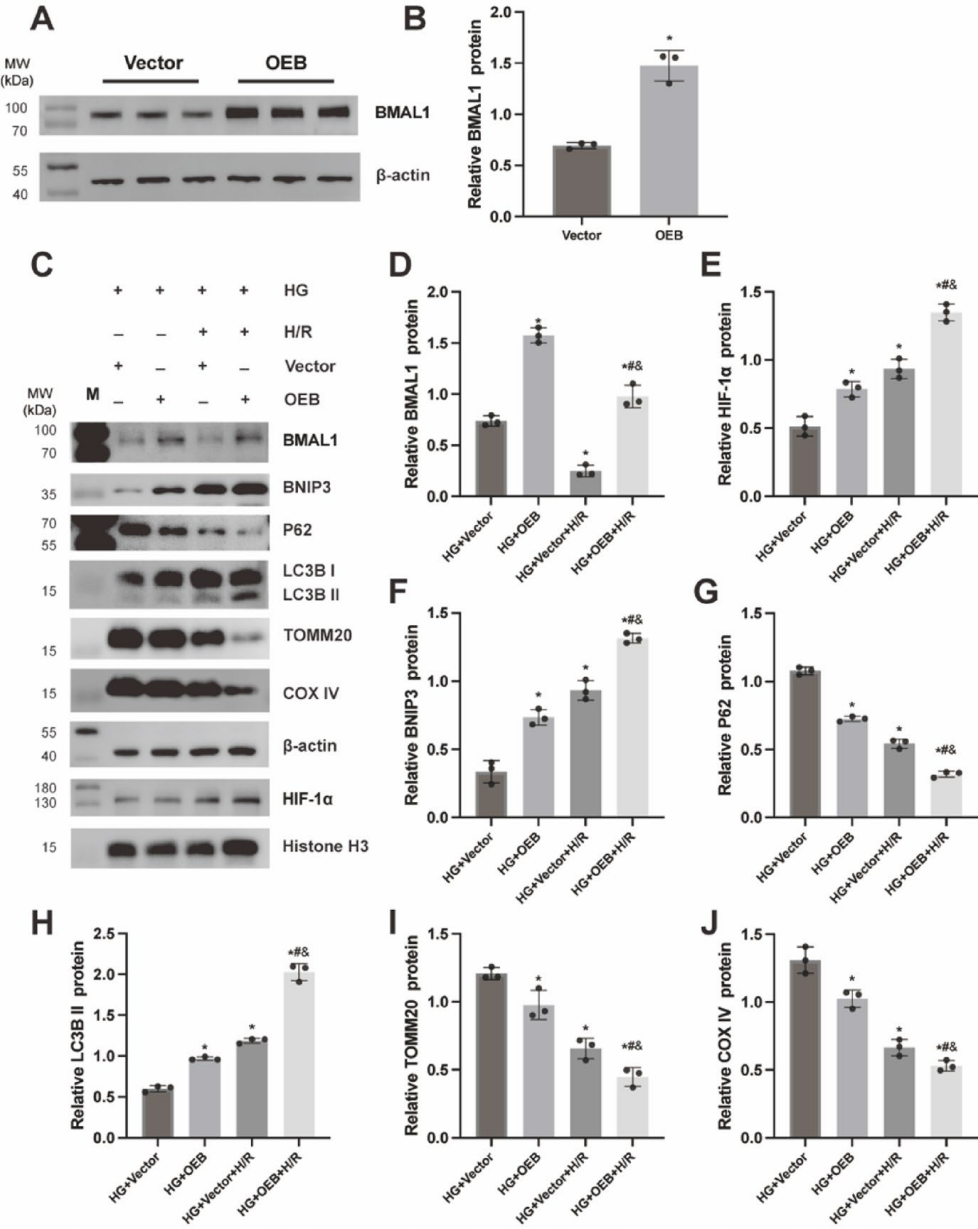
Overexpression of BMAL1 induces mitophagy mediated by the HIF-1α/BNIP3 pathway. TCMK-1 cells were transfected with BMAL1-OE lentiviral vector and BMAL1-NC empty vector to construct the BMAL1 overexpression group (OEB group) and the empty vector control group (Vector group) respectively. Cells from each group were collected, and WB was performed to detect the expression of BMAL1, HIF-1α, BNIP3, p62, LC3B I, LC3B II, TOMM20, and COX IV proteins. (**A**,** B**) WB image and BMAL1 expression of vector and OEB group. (**C**) WB image. (**D**-**J**) Statistical analysis of BMAL1, HIF-1α, BNIP3, p62, LC3B II, TOMM20, and COX IV expression. Results are expressed as means ± SD; *n* = 3 independent experiments, otherwise specified. *P < 0.05 vs. the HG + Vector group; #*P* < 0.05 vs. the HG + OEB group; &*P* < 0.05 vs. the HG + Vector + H/R group. Supplementary Fig. S5 includes both cropped images and full-length blots.

To elucidate the impact of BMAL1 on mitophagy through the HIF-1α/BNIP3 pathway under HG and H/R conditions, we transfected TCMK-1 cells with an BMAL1-OE lentiviral vector to generate stable BMAL1-overexpressing cells (OEB group), using the BMAL1-NC empty vector as a negative control (Vector group). As illustrated in Fig. 5, BMAL1 expression was markedly higher in the OEB group compared to the Vector group. This upregulation was accompanied by increased levels of HIF-1α, BNIP3, and LC3B II, while levels of p62, TOMM20, and COX IV were notably decreased. These findings indicate that BMAL1 overexpression augments mitophagy through the HIF-1α/BNIP3 signaling pathway.

### Overexpression of BMAL1 alleviates TCMK-1 cell injury and mitochondrial dysfunction induced by HG and H/R

**Fig. 6:**
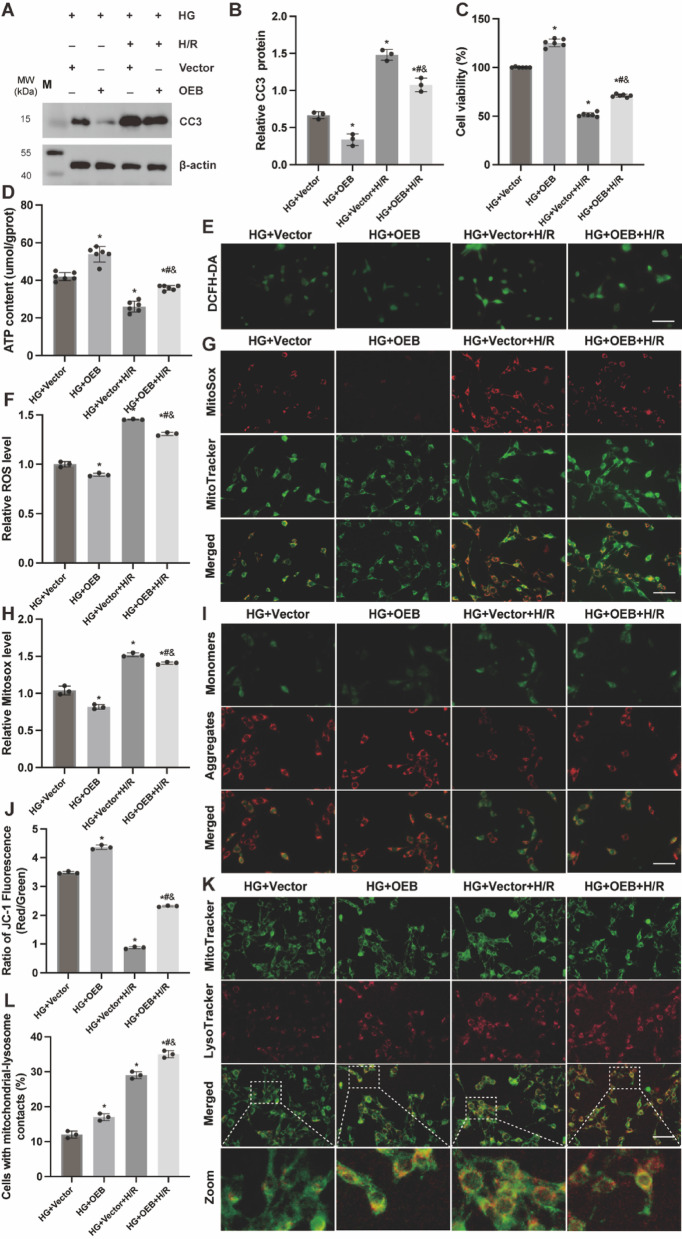
Overexpression of BMAL1 alleviates TCMK-1 cell injury and mitochondrial dysfunction induced by HG and H/R. (**A**,** B**) WB analysis of CC3 protein expression levels in kidney tissue. (**C**) Cell viability detected by CCK8 assay, *n* = 6 independent experiments. (**D**) ATP content, *n* = 6 independent experiments. (**E**,** F**) ROS levels detected by DCFH-DA probe. (**G**,** H**) Mitochondrial ROS levels detected by MitoSox Red. (**I**) JC-1 staining observation of mitochondrial membrane potential. (**J**) JC-1 staining analysis of the ratio of green/red fluorescence intensity, reflecting MMP levels. (**K**) Representative fluorescent images of lysophagy in each group. (**L**) Quantification of K. Scale bar = 50 μm. Results are expressed as means ± SD; *n* = 3 independent experiments, otherwise specified. ^*^P < 0.05 vs. the HG + Vector group; ^#^*P* < 0.05 vs. the HG + OEB group; ^&^*P* < 0.05 vs. the HG + Vector + H/R group. Supplementary Fig. S6 includes both cropped images and full-length blots.

To elucidate the impact of BMAL1 expression on TCMK-1 cells subjected to HG conditions during H/R injury, we evaluated alterations in cell viability, apoptosis, and mitochondrial function. Both HG and H/R treatments led to reduced cell viability and ATP production, alongside increased apoptosis. However, BMAL1 overexpression mitigated these effects, restoring cell viability and ATP levels while decreasing apoptosis (Figs. 6A, B, C, D). Additionally, elevated BMAL1 expression was associated with a reduction in ROS generation and attenuation of MMP damage induced by HG and H/R, as well as an increase in mitochondrial-lysosomal fusion events (Figs. 6E-L). These results suggest that BMAL1 overexpression ameliorates cellular injury and mitochondrial dysfunction in the context of HG and H/R exposure. These results suggest that under HG and H/R exposure, BMAL1 overexpression can alleviate cellular damage and mitochondrial dysfunction and partially restore the level of mitophagy.

### The mitophagy mediated by the HIF-1α/BNIP3 pathway is attenuated by the HIF-1α inhibitor PX-478

**Fig. 7:**
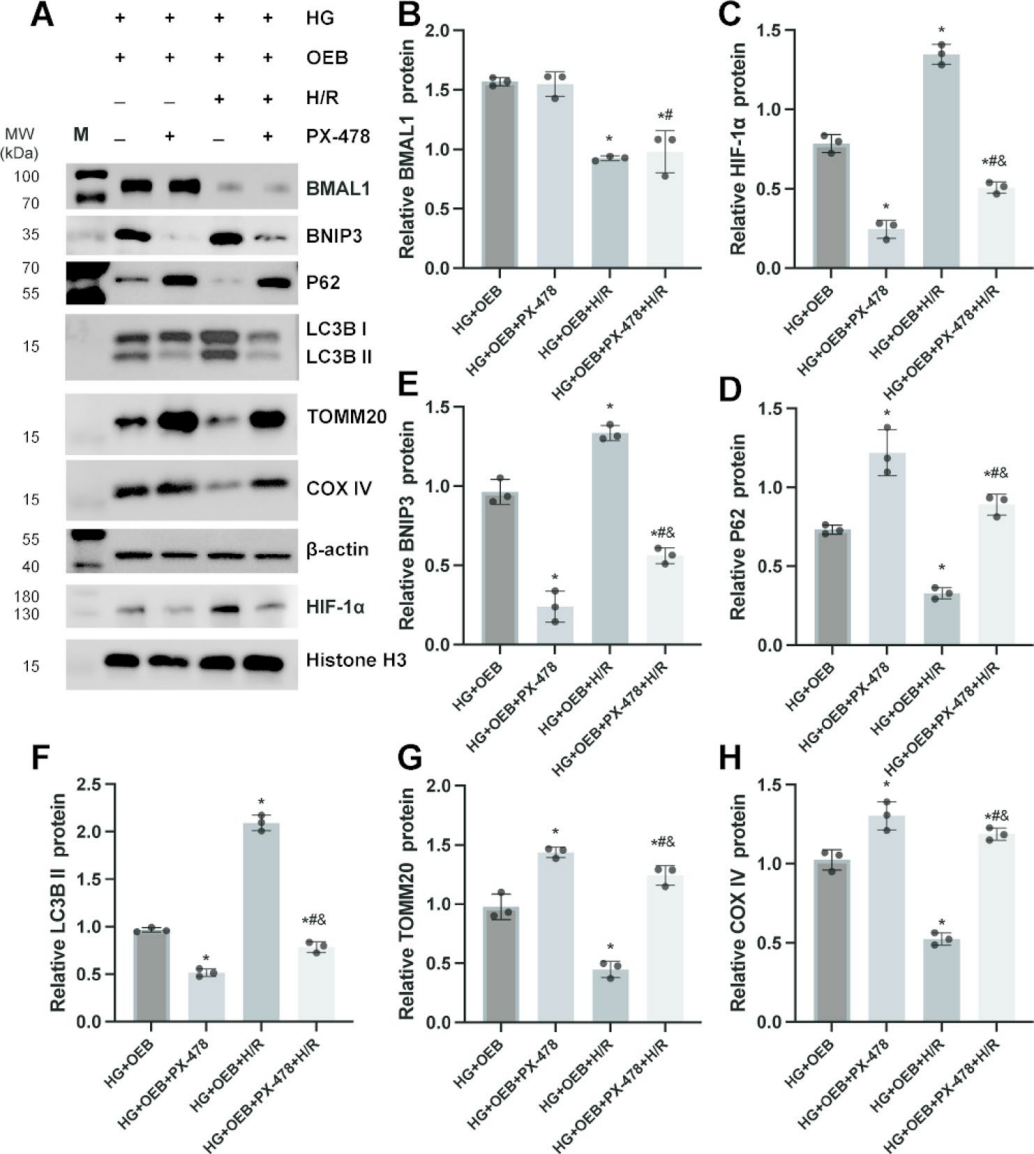
The mitophagy mediated by the HIF-1α/BNIP3 pathway is attenuated by the HIF-1α inhibitor PX-478 (10µM). Cells from each group were collected, and WB was performed to detect the protein expression of HIF-1α, BNIP3, p62, LC3B I, LC3B II, TOMM20, and COX IV. (**A**) WB images. (**B**-**H**) Statistical analysis of the expression of BMAL1, HIF-1α, BNIP3, p62, LC3B II, TOMM20, and COX IV. Results are expressed as means ± SD; *n* = 3 independent experiments, otherwise specified. ^*^P < 0.05 vs. the HG + OEB group; ^#^*P* < 0.05 vs. the HG + OEB + PX478 group; ^&^*P* < 0.05 vs. the HG + OEB + H/R group. Supplementary Fig. S7 includes both cropped images and full-length blots.

To further explore whether BMAL1 overexpression enhances mitophagy through the HIF-1α/BNIP3 pathway, we treated cells with the HIF-1α inhibitor PX-478 (10 µM). As shown in Fig. 7, the PX-478-treated group exhibited a marked reduction in HIF-1α levels compared to both the HG + OEB and HG + OEB + H/R groups. This decrease in HIF-1α was accompanied by diminished levels of BNIP3 and LC3B II. In contrast, there was a notable increase in the expression of p62, TOMM20, and COX IV, suggesting that the inhibitory effect of PX-478 on HIF-1α impairs the BMAL1-induced mitophagy. It is worth noting that PX-478 has no significant effect on the expression of BMAL1.

### The protective effect of BMAL1 on cell damage and mitochondrial function is attenuated by the HIF-1α inhibitor PX-478

**Fig. 8:**
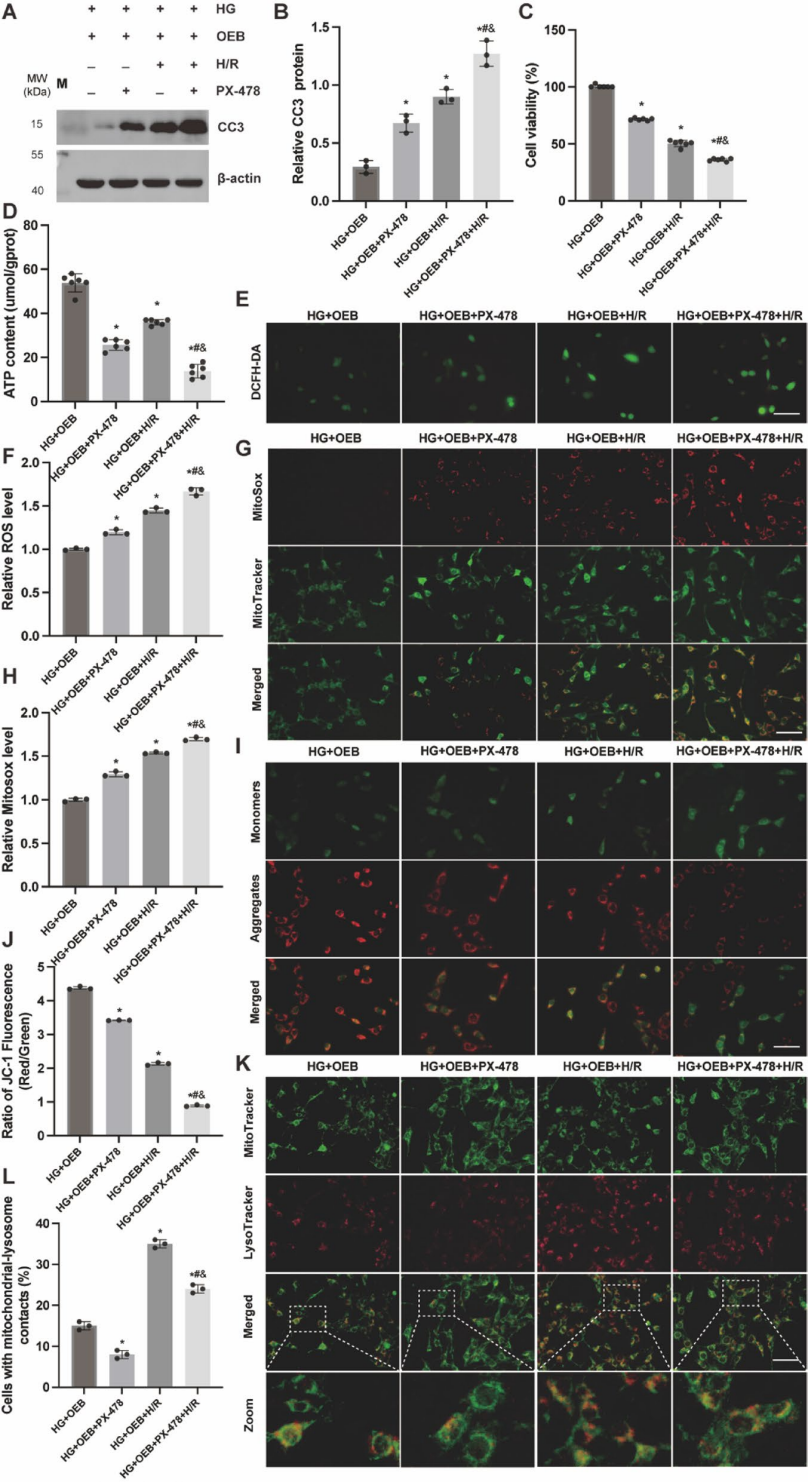
The protective effect of BMAL1 on cell damage and mitochondrial function is attenuated by the HIF-1α inhibitor PX-478. (**A**,** B**) WB analysis of CC3 protein expression levels in kidney tissue. (**C**) Cell viability detected by CCK8 assay, *n* = 6 independent experiments. (**D**) ATP content, *n* = 6 independent experiments. (**E**,**F**) ROS levels detected by DCFH-DA probe. (**G**,** H**) Mitochondrial ROS levels detected by MitoSox Red. (**I**) JC-1 staining observation of mitochondrial membrane potential. (**J**) JC-1 staining analysis of the ratio of green/red fluorescence intensity, reflecting MMP levels. (**K**) Representative fluorescent images of lysophagy in each group. (**L**) Quantification of K. Scale bar = 50 μm. Results are expressed as means ± SD; *n* = 3 independent experiments, otherwise specified. ^*^P < 0.05 vs. the HG + OEB group; ^#^*P* < 0.05 vs. the HG + OEB + PX478 group; ^&^*P* < 0.05 vs. the HG + OEB + H/R group. Supplementary Fig. S8 includes both cropped images and full-length blots.

To explore whether BMAL1 overexpression facilitates mitophagy through the HIF-1α/BNIP3 pathway to protect cells under high glucose and hypoxia-reoxygenation conditions, we re-evaluated cell viability, CC3 expression, and mitochondrial function. As illustrated in Fig. 8, cells in the PX-478 group exhibited reduced viability and ATP content, alongside increased ROS production, compromised MMP, decreased mitochondrial-lysosomal fusion events, and exacerbated apoptosis. These findings suggest that BMAL1 overexpression mitigates cellular damage induced by high glucose and hypoxia-reoxygenation by enhancing HIF-1α/BNIP3-mediated mitophagy.

## Discussion

Diabetes is a group of chronic metabolic disorders marked by persistent hyperglycemia, which can lead to multi-organ complications, including cardiovascular abnormalities and renal failure^25^. Diabetic nephropathy is the predominant chronic kidney ailment, serving as a significant contributor to end-stage renal failure^26^. Accumulating data showing that diabetic kidneys exhibit increased susceptibility to I/RI^1-3^, which is closely related to mitophagy^18^. The involvement of the circadian gene BMAL1 in regulating renal I/RI has gained recognition in recent studies^13-16^. However, its specific role and underlying mechanisms in diabetic renal I/RI remain unclear. To address this, we sought to explore how BMAL1 modulates mitophagy in the context of diabetic renal I/RI through both in vivo and in vitro experiments. Our findings indicate that the reduced expression of BMAL1 in diabetic kidneys is a critical contributor to the increased susceptibility to diabetic renal I/RI. In contrast, BMAL1 overexpression appears to provide renal protection in diabetes by promoting mitophagy via the HIF-1α/BNIP3 signaling pathway.

Renal tubular epithelial cells are rich in mitochondria and highly sensitive to ischemia^27^. Under ischemic conditions, the kidney shifts from aerobic metabolism to anaerobic metabolism, significantly reducing the cells’ ability and rapidly regenerate ATP. Simultaneously, Mitochondrial damage results in elevated ROS production, which promotes the release of cytochrome c and other pro-apoptotic factors from the mitochondria into the cytoplasm, thereby triggering the cell death pathway^28,29^. Mitochondrial autophagy is a critical factor in maintaining mitochondrial homeostasis and protecting normal kidney function. During the I/RI process, mitophagy is activated as an endogenous protective mechanism, selectively clearing damaged or dysfunctional mitochondria by encapsulating them into autophagosomes and promoting their fusion with lysosomes for mitochondrial degradation, thereby maintaining cellular homeostasis. By removing damaged mitochondria, mitochondrial autophagy can reduce the production of ROS and oxidative stress damage^30^. Additionally, mitochondrial autophagy inhibits the activation of caspase-3 and reduces apoptosis of renal tubular epithelial cells, thereby alleviating renal ischemia-reperfusion injury^21,31^. BNIP3 is a key protein in the receptor-dependent pathway of mitophagy, playing a crucial role in maintaining normal cellular function^32^. It is a critical regulatory factor in mitochondrial quality control and the process of apoptosis^33,34^. Its regulation of mitochondrial fragmentation and mitophagy is achieved through interactions with LC3 (microtubule-associated protein 1 light chain 3) and its associated receptors^35,36^. In renal tubular cells, I/RI induces activation of mitophagy and upregulation of BNIP3 expression. Silencing or knockout of BNIP3 results in reduced levels of mitochondrial autophagy, increased ROS levels, enhanced apoptosis, and exacerbation of kidney I/RI^37^. Conversely, overexpression of BNIP3 increases mitochondrial autophagy levels and mitigates kidney I/RI damage, indicating that BNIP3 plays a renal protective role by regulating mitochondrial autophagy in renal tubular cells^38^. HIF-1α is an upstream regulator of BNIP3, which can directly increase the transcription and expression of BNIP3^39^. BNIP3 is upregulated in a HIF-1α-dependent manner during renal I/RI^21,37^. In alignment with these findings, our data demonstrate that renal I/RI triggers an increase in HIF-1α, which subsequently drives mitophagy through BNIP3. However, diabetes might exacerbate renal I/RI by suppressing mitophagy^18^. Consistent with these findings, our study shows that db/db mice exhibit increased susceptibility to renal ischemia-reperfusion injury, accompanied by a reduction in mitochondrial autophagy levels in renal tissues. Our in vitro experimental results further confirm this.

The kidney’s physiological processes, including renal blood flow, glomerular filtration rate, and hormone secretion, exhibit significant circadian rhythmic changes^40^. BMAL1, a key regulator of the circadian clock, also exhibits circadian rhythmic characteristics in its expression in the kidney^6,7^. In the present study, efforts were made to keep the modeling and sampling times consistent among groups to minimize the influence of circadian rhythmic changes in BMAL1 expression upon the experimental results. Notably, the absence or dysfunction of BMAL1 is recognized as a critical factor in the onset and progression of diabetic nephropathy^9,11^. Emerging evidence highlights a strong link between circadian rhythms and mitochondrial homeostasis, with mitochondrial dynamics and mitophagy being regulated by clock genes^41,42^, while activation of mitophagy can mitigate ischemia-reperfusion-induced renal damage^18,43^. Consistent with these findings, our study indicates that overexpression of BMAL1 was able to alleviate the inhibitory effect of high glucose on mitophagy via the HIF-1α/BNIP3 signaling pathway, thereby reducing mitochondrial dysfunction and cell damage caused by hypoxia-reoxygenation. However, the precise mechanisms through which BMAL1 influences the HIF-1α/BNIP3 pathway remain unclear.

Recent studies have revealed that the bHLH-PAS transcription factor superfamily, which includes both HIF-1α and BMAL1, plays a critical role in sensing and responding to physiological and environmental cues. Notably, the HIF-1α mRNA transcripts also exhibit diurnal rhythmic oscillations^44^, suggesting a close relationship between HIF-1α and BMAL1. The potential mechanisms by which BMAL1 regulates HIF-1α can be analyzed from multiple aspects, including transcription, post-translational modifications, metabolic regulation, and its interaction with other molecular pathways. Studies suggest that BMAL1 may directly participate in the transcriptional regulation of the HIF-1α gene by binding to E-box elements^24,45^. BMAL1 may also indirectly regulate its expression by affecting the activity of other transcription factors related to the HIF-1α gene, such as CLOCK^46^. On the level of post-translational modifications, BMAL1 may indirectly influence the hydroxylation level of HIF-1α by regulating the activity of oxygen-sensitive PHD enzymes, thereby modulating its stability at the protein level^47^. Additionally, BMAL1 may act by affecting hypoxic signaling pathways, which influence HIF-1α nuclear translocation or ubiquitination-mediated degradation^44^. BMAL1 can also indirectly affect HIF-1α activity by regulating cellular metabolism, such as glycolysis and oxidative phosphorylation^24^. Furthermore, BMAL1 can cooperate with other transcription factors, such as CLOCK or RORα, to regulate the activity of HIF-1α^48^. BMAL1 may also affect its transcriptional activity in the nucleus by modulating its interaction with other coactivators, such as p300/CBP, thereby altering the expression pattern of HIF-1α target genes^47,48^. In this study, the high-glucose environment led to further downregulation of BMAL1 expression, which may reduce HIF-1α protein levels either by directly regulating HIF-1α transcription or by affecting its stability. This, in turn, inhibits mitochondrial autophagy mediated by the HIF-1α/BNIP3 pathway and exacerbates renal injury induced by ischemia-reperfusion.

Research has demonstrated that HIF-1α exerts a protective effect in renal I/RI^19,49^. However, under diabetic conditions, the hypoxia-induced adaptive response of HIF-1α is significantly impaired^50^, with elevated glucose levels notably suppressing HIF-1α expression in renal proximal tubular epithelial cells during hypoxia^51^. This study reveals that BMAL1 overexpression, in the context of high glucose levels, effectively restores the responsiveness of HIF-1α to hypoxia-reoxygenation injury. This restoration enhances mitophagy and alleviates oxidative stress. Nevertheless, the protective effects of increased BMAL1 on TCMK-1 cells are significantly reduced by the HIF-1α inhibitor PX-478. PX-478, as a HIF-1α inhibitor, primarily reduces HIF-1α protein levels through three mechanisms, with the reduction of HIF-1α mRNA levels and inhibition of HIF-1α translation playing the key roles. Additionally, PX-478 treatment can inhibit the deubiquitination of HIF-1α, thereby increasing the levels of polyubiquitinated HIF-1α^52^. Overexpression of BMAL1 alleviates cell damage caused by high glucose and hypoxia-reoxygenation by enhancing mitochondrial autophagy, whereas PX-478 inhibits HIF-1α, leading to a decrease in BNIP3 expression, thereby reducing mitochondrial autophagy levels. This results in the accumulation of damaged mitochondria, which exacerbates cell injury and compromises the protective effect of BMAL1.This observation indicates that BMAL1’s promotion of mitophagy is largely mediated through the activation of HIF-1α.

In conclusion, our results demonstrate that diminished expression of the clock gene BMAL1 markedly increases susceptibility to diabetic kidney I/RI. Conversely, elevated levels of BMAL1 facilitate mitophagy via the HIF-1α/BNIP3 signaling pathway, thereby alleviating mitochondrial dysfunction, cellular damage, and apoptosis. Future investigations will explore the intricate interplay between circadian rhythms and the HIF-1α/BNIP3 signaling pathway. By incorporating the cyclical fluctuations of circadian genes, we aim to construct models that capture these interactions throughout different phases of the circadian cycle. Furthermore, we will develop murine models with BMAL1 overexpression and HIF-1α knockout mice to elucidate the underlying mechanisms. These investigations may pave the way for novel therapeutic approaches to treat renal I/RI in diabetic patients.

## Methods

### Cell culture

The tubular epithelial cell in mouse (TCMK-1 cells), from the Cell Bank of the Chinese Academy of Sciences (Shanghai, China), were cultured in Minimum Essential Medium (MEM, Hyclone, Beijing, China) containing 10% fetal bovine serum (FBS, Bioexplorer, USA) in the incubator with 5% CO2 at 37 °C.

### TCMK-1 cell high glucose model and hypoxia/reoxygenation model

In the H/R group, cells were first incubated in serum-free MEM medium for 12 h, followed by exposure to MEM medium supplemented with 10% FBS under hypoxic conditions (94% N2 + 5% CO2) for 24 h. Subsequently, the cells were transferred to a standard incubator with 5% CO2 for 12 h of reoxygenation. In contrast, the NC group cells were continuously maintained in a standard incubator. The HG group cells were cultured in MEM medium containing high glucose (30 mM).

### Stable overexpression of BMAL1

The lentivirus was purchased from OBiO Technology(Shanghai). The lentiviral expression vector designated BMAL1-OE(pSLenti-EF1-P2A-Puro-CMV-ARNTL-3xFlag-WPRE) was used for BMAL1 gene (NM_001297719.2) delivery and stable overexpression. The empty vector designated BMAL1-NC (pSLenti-EF1-P2A-Puro-CMVMCS-3xFlag-WPRE) was used as a negative control. The MOI was 20. According to the manufacturer’s protocol, puromycin (50 µg/ml) was used to screen uninfected cells, and the surviving cells were further cultured and expanded. The successful overexpression of BMAL1 was subsequently validated through Western blot analysis.

### Preparation and administration of HIF-1α inhibitor PX-478

The HIF-1α inhibitor PX-478 (S-2-amino-3-[4′-N, N,-bis(chloroethyl)amino]phenyl propionic acid N-oxide dihydrochloride, HY-10231, MedChemExpress, Shanghai, China) was prepared by dissolving 5 mg of PX-478 in 1 mL of dimethyl sulfoxide (DMSO; Sigma-Aldrich, USA) to create a stock solution. Subsequently, 40 µL of this stock solution was diluted into 50 mL of culture medium, resulting in a final concentration of 10 µM PX-478.

### Experimental animals

All animal experiments conducted in this study adhered to the Laboratory Animal Care guidelines set forth by Wuhan University and received approval from the Committee for the Use of Live Animals in Teaching and Research. Additionally, the study is reported in accordance with ARRIVE guidelines^53^. We procured twelve eight-week-old male db/db (BKS.Cg-Dock7m+/+Leprdb/J) mice and twelve eight-week-old male non-diabetic (db/+) mice from Shulaibao (Wuhan) Biotechnology Co., Ltd. The mice were maintained at the Animal Experimental Center of Renmin Hospital, Wuhan University, under controlled environmental conditions, including a temperature of 22 ± 2 °C and regulated humidity. They were kept on a 12-hour light/dark cycle.

### Renal I/R injury model

Bilateral renal I/R injury was induced in anesthetized mice. Briefly, mice were first weighed and then anesthetized with an intraperitoneal injection of pentobarbital sodium (50 mg/kg)^54^. A mid-abdominal incision was made to expose the bilateral renal arteries, which were then clamped for 30 min to induce ischemia. After this period, blood flow was restored to the kidneys, and the mice were euthanized by using an excessive amount of anesthetic to collect blood and tissue samples for subsequent analyses. Mice with unclamped renal arteries served as controls for the no I/R condition.

### Experimental protocols

For the in vivo studies, db/+ mice were allocated into two groups: the normal sham (NS group, *n* = 6) and the normal ischemia/reperfusion (NI/R group, *n* = 6). Similarly, db/db mice were divided into the diabetes sham (DS group, *n* = 6) and the diabetes ischemia/reperfusion (DI/R group, *n* = 6).

In the in vitro experiments, TCMK-1 cells were assigned to one of four conditions: negative control (NC group), high glucose control (HG group), negative control with hypoxia/reoxygenation (NH/R group), and high glucose with hypoxia/reoxygenation (HG + H/R group). Cells were further categorized based on the presence of BMAL1 overexpression lentivirus transfection into four distinct groups: high glucose with empty vector control (HG + Vector group), high glucose with BMAL1 overexpression (HG + OEB group), high glucose with empty vector control and hypoxia/reoxygenation (HG + Vector + H/R group), and high glucose with BMAL1 overexpression and hypoxia/reoxygenation (HG + OEB + H/R group). Additionally, the effect of PX-478 treatment was assessed by dividing cells into: high glucose with BMAL1 overexpression and no PX-478 (HG + OEB group), high glucose with BMAL1 overexpression and PX-478 (HG + OEB + PX-478 group), high glucose with BMAL1 overexpression and hypoxia/reoxygenation (HG + OEB + H/R group), and high glucose with BMAL1 overexpression, PX-478, and hypoxia/reoxygenation (HG + OEB + PX-478 + H/R group).

### Cell viability assay

Cell viability was evaluated using the CCK-8 assay (CK04, Dojindo, Kumamoto, Japan) following the manufacturer’s protocol. Briefly, TCMK-1 cells were seeded in 96-well plates at 5 × 10^3^ cells per well. After exposure to hypoxia/reoxygenation, 10 µL of CCK-8 solution was added to each well, and cells were incubated for 3 h at 37 °C. Hydrogen peroxide (H_2_O_2_) was used as a positive control to induce cytotoxicity, with untreated cells serving as a negative control (Figure S9). Absorbance at 450 nm was subsequently measured with a microplate reader to determine cell viability.

### Determination of intracellular ROS and mitochondrial ROS

Intracellular ROS in TCMK-1 cells were quantified using dichloro-dihydro-fluorescein diacetate (DCFH-DA). TCMK-1 cells were seeded in 6-well plates at 2 × 10^5^ cells per well. The cells in each well were incubated with 2 mL of 10 µM DCFH-DA (S0033, Beyotime, Shanghai, China) for 30 min at 37 °C in darkness, followed by three washes with serum-free medium. The same method was then employed to measure mitochondrial ROS, substituting DCFH-DA probe with MitoSOX Red (5 µM, MCE, USA). We applied treatments with H_2_O_2_ to increase ROS levels significantly, and used untreated samples as negative controls to demonstrate baseline ROS levels (Figure S9). Fluorescence images were captured using an Olympus fluorescence microscope (Tokyo, Japan). The relative fluorescence intensity was assessed using ImageJ software (Version 1.53).

### Measurement of intracellular ATP

ATP content was quantified using an ATP microplate assay kit (abs580117, Absin, Shanghai, China) following the manufacturer’s guidelines. A reduction in ATP levels serves as an indicator of cellular injury, reflecting potential mitochondrial dysfunction or impairment. Oligomycin was used as a positive control to inhibit ATP synthase and decrease ATP levels, alongside untreated samples as negative controls (Figure S9).

### Assessment of mitochondrial membrane potential with JC-1 staining

MMP was evaluated using a JC-1 MMP assay kit (C2003, Beyotime), following the manufacturer’s protocol. TCMK-1 cells were seeded in 6-well plates at 2 × 10^5^ cells per well. The cells in each well were treated with 1 ml of a JC-1 staining solution, prepared by diluting 5 µl of a 200X JC-1 stock solution with 1 ml of JC-1 staining buffer. This mixture was incubated for 20 min at 37 °C in the dark. Following incubation, cells were washed twice with JC-1 staining buffer. Carbonyl cyanide m-chlorophenyl hydrazone (CCCP) was used to induce mitochondrial depolarization as a positive control, with untreated cells serving as a negative control (Figure S9). Fluorescence microscopy was employed to assess the results. Elevated MMP is indicated by JC-1 aggregation within the mitochondrial matrix, resulting in red fluorescence, whereas diminished MMP is marked by the inability of JC-1 to aggregate, producing green fluorescence due to its monomeric form. MMP changes were quantified based on the relative intensity of red and green fluorescence.

### MitoTracker and lysotracker

After special treatment, the cells were labelled with mitochondria and lysosomes, which were tracked using commercial Mito-Tracker Green (C1048, Beyotime, Shanghai, China) and Lyso-Tracker Red (C1046, Beyotime, Shanghai, China), according to the manufacturer’s instructions.

### Serum assays

SCr and BUN levels were quantified using a creatinine assay kit (EICT-100, BioAssay Systems) and a urea assay kit (DIUR-100, BioAssay Systems), respectively, following the manufacturer’s instructions.

### Histopathological evaluation of kidney injury

Kidney samples were fixed in 4% paraformaldehyde, embedded in paraffin, and sectioned into 4 μm-thick slices. These sections were then stained with hematoxylin and eosin (HE). Tissue pathology was evaluated using an upright microscope, and tubular injury severity was quantified using the Paller renal tubular injury scoring system [28]. This system involves the random selection of 10 renal tubules per high-power field. Scores were assigned based on the following criteria: 1 point for tubular dilation, cell flattening, or swelling; 1 or 2 points for brush border injury or detachment; 2 points for the presence of casts; and 1 point for detached necrotic cells within the tubular lumen, provided these did not form casts or fragments.

### Western blotting

For the detection of protein levels, the total protein was extracted using RIPA Lysis Buffer (GB2002, Servicebio, Wuhan, China) supplemented with a cocktail of protease inhibitor (GB2006, Servicebio, Wuhan, China), phosphatase inhibitor (GB2007, Servicebio, Wuhan, China), and phenylmethylsulfonyl fluoride (GB2008, Servicebio, Wuhan, China). The nuclear protein was isolated using NE-PER Nuclear and Cytoplasmic Extraction Reagents (Thermo Scientific, 78833) to detect HIF-1α expression. Protein extracts from cells or renal cortical tissues were quantified using the BCA reagent (P0010S, Beyotime Biotechnology). Following separation by sodium dodecyl sulfate-polyacrylamide gel electrophoresis (SDS-PAGE), the proteins were transferred to a polyvinylidene difluoride membrane (Millipore, Billerica, MA, USA). The membrane was then blocked with 5% skim milk for 60 min. Subsequently, the membrane was incubated with primary antibodies, followed by horseradish peroxidase-conjugated secondary antibodies (AS003, AS014, Abclonal, Wuhan, China). The primary antibodies used were diluted as follows: HIF-1α (GB114936-100, Servicebio, Wuhan, China) at 1:1000, Histone H3 (ab1791, Abcam, Cambridge, FL, USA) at 1:5000, BMAL1 (14020, Cell Signaling Technology, Danvers, MA, USA) at 1:1000, LC3B (83506, Cell Signaling Technology, Danvers, MA, USA) at 1:1000, COX IV (4850, Cell Signaling Technology, Danvers, MA, USA) at 1:500, TOMM20 (42406, Cell Signaling Technology, Danvers, MA, USA) at 1:1000, p62 (39749, Cell Signaling Technology, Danvers, MA, USA) at 1:500, BNIP3 (ab109362, Abcam, Cambridge, FL, USA) at 1:1000, cleaved caspase 3 (CC3) (9661, Cell Signaling Technology, Danvers, MA, USA) at 1:1000, and β-actin (GB15001-100, Servicebio, Wuhan, China) at 1:3000. β-actin or Histone H3 was used as a loading control to ensure equal protein loading. Protein expression levels were quantified based on gray value analysis using ImageJ software (Version 1.53).

###  Determination of apoptosis

To assess apoptosis, paraffin-embedded renal sections were subjected to staining with a TUNEL kit (Roche, Basel, Switzerland), followed by counterstaining with 4′,6-diamidino-2-phenylindole (DAPI) for nuclear visualization. Apoptosis was quantified in accordance with the manufacturer’s guidelines. For each sample, ten random fields were examined to determine the apoptosis index.

### Transmission electron microscopy (TEM) analysis

Upon completion of the mouse modeling, the kidneys were harvested. Thin tissue slices, obtained from the interface of the renal cortex and medulla on ice, were immediately fixed in 2.5% glutaraldehyde. Following fixation, the samples were post-fixed in 1% osmium tetroxide, dehydrated through a graded ethanol series, and embedded in a hard resin. The embedded samples were then sectioned into ultra-thin slices of 80 nm. These sections were subjected to double staining with uranyl acetate and lead citrate, and subsequently examined using a transmission electron microscope (H-600, Hitachi) to evaluate mitochondrial morphology and quality. For all cases, 5–6 images were analyzed per sample and mitochondrial morphology per field were calculated. Each image represents 2–3 cells per field. Each cell contained ~ 50–100 mitochondria. Mitochondria length was obtained using the major axis as reported previously^55^. TEM images were analyzed utilizing ImageJ software (Version 1.53).

### Statistical analysis

Data are presented as mean ± SD. Statistical analysis was performed using GraphPad Prism software (Version 9.4.1, USA). Comparisons among multiple groups were conducted using one-way analysis of variance (ANOVA), with Tukey’s test employed for pairwise comparisons. A p-value of less than 0.05 was deemed statistically significant.

## Electronic supplementary material

Below is the link to the electronic supplementary material.

Supplementary Material 1

## Data Availability

The data supporting the findings of this study are available within the manuscript or supplementary information files. Should any raw data files be needed in another format they are available from the corresponding author upon reasonable request.

## References

[1] Muroya, Y. et al. Enhanced renal ischemia-reperfusion injury in aging and diabetes. *Am. J. Physiol. Ren. Physiol.* **315**, F1843–f1854. 10.1152/ajprenal.00184.2018 (2018). 10.1152/ajprenal.00184.2018 PMC6336981 30207168

[2] de Ponte, M. C., Cardoso, V. G., Gonçalves, G. L., Costa-Pessoa, J. M. & Oliveira-Souza, M. Early type 1 diabetes aggravates renal ischemia/reperfusion-induced acute kidney injury. *Sci. Rep.* **11**, 19028. 10.1038/s41598-021-97839-7 (2021). 34561469 10.1038/s41598-021-97839-7 PMC8463569

[3] Gong, D. J., Wang, L., Yang, Y. Y., Zhang, J. J. & Liu, X. H. Diabetes aggravates renal ischemia and reperfusion injury in rats by exacerbating oxidative stress, inflammation, and apoptosis. *Ren. Fail.* **41**, 750–761. 10.1080/0886022x.2019.1643737 (2019). 31441362 10.1080/0886022X.2019.1643737 PMC6720228

[4] Han, S. J. & Lee, H. T. Mechanisms and therapeutic targets of ischemic acute kidney injury. *Kidney Res. Clin. Pract.* **38**, 427–440. 10.23876/j.krcp.19.062 (2019). 31537053 10.23876/j.krcp.19.062 PMC6913588

[5] Mohawk, J. A., Green, C. B. & Takahashi, J. S. Central and peripheral circadian clocks in mammals. *Annu. Rev. Neurosci.* **35**, 445–462. 10.1146/annurev-neuro-060909-153128 (2012). 22483041 10.1146/annurev-neuro-060909-153128 PMC3710582

[6] Cox, K. H. & Takahashi, J. S. Circadian clock genes and the transcriptional architecture of the clock mechanism. *J. Mol. Endocrinol.* **63**, R93–r102. 10.1530/jme-19-0153 (2019). 31557726 10.1530/JME-19-0153 PMC6872945

[7] Zhang, D. & Pollock, D. M. Circadian regulation of kidney function: finding a role for Bmal1. *Am. J. Physiol. Ren. Physiol.* **314**, F675–F678. 10.1152/ajprenal.00580.2017 (2018). 10.1152/ajprenal.00580.2017 PMC6031908 29357439

[8] Ansermet, C. et al. Dysfunction of the circadian clock in the kidney tubule leads to enhanced kidney gluconeogenesis and exacerbated hyperglycemia in diabetes. *Kidney Int.* **101**, 563–573. 10.1016/j.kint.2021.11.016 (2022). 34838539 10.1016/j.kint.2021.11.016

[9] Peng, Z. et al. New insights into the mechanisms of diabetic kidney disease: role of circadian rhythm and Bmal1. *Biomed. Pharmacother*. **166**, 115422. 10.1016/j.biopha.2023.115422 (2023). 37660646 10.1016/j.biopha.2023.115422

[10] Su, W. et al. Altered clock gene expression and vascular smooth muscle diurnal contractile variations in type 2 diabetic Db/db mice. *Am. J. Physiol. Heart Circ. Physiol.* **302**, H621–633. 10.1152/ajpheart.00825.2011 (2012). 22140039 10.1152/ajpheart.00825.2011 PMC3353796

[11] Marcheva, B. et al. Disruption of the clock components CLOCK and BMAL1 leads to hypoinsulinaemia and diabetes. *Nature* **466**, 627–631. 10.1038/nature09253 (2010). 20562852 10.1038/nature09253 PMC2920067

[12] Wang, Y., Cai, J., Tang, C. & Dong, Z. Mitophagy in acute kidney injury and kidney repair. *Cells* **9**10.3390/cells9020338 (2020). 10.3390/cells9020338 PMC7072358 32024113

[13] Dong, C. et al. Denervation aggravates renal ischemia reperfusion injury via BMAL1-mediated Nrf2/ARE pathway. *Arch. Biochem. Biophys.* **746**, 109736. 10.1016/j.abb.2023.109736 (2023). 37657745 10.1016/j.abb.2023.109736

[14] Sun, Q., Zeng, C., Du, L. & Dong, C. Mechanism of circadian regulation of the NRF2/ARE pathway in renal ischemia-reperfusion. *Exp. Ther. Med.* **21**, 190. 10.3892/etm.2021.9622 (2021). 33488799 10.3892/etm.2021.9622 PMC7812573

[15] Ville, S. et al. Timing of kidney clamping and deceased donor kidney transplant outcomes. *Clin. J. Am. Soc. Nephrol.* **16**, 1704–1714. 10.2215/cjn.03290321 (2021). 34625421 10.2215/CJN.03290321 PMC8729417

[16] Ye, P. et al. BMAL1 regulates mitochondrial homeostasis in renal ischaemia-reperfusion injury by mediating the SIRT1/PGC-1α axis. *J. Cell. Mol. Med.* **26**, 1994–2009. 10.1111/jcmm.17223 (2022). 35174626 10.1111/jcmm.17223 PMC8980910

[17] Granata, S. et al. Oxidative stress and ischemia/reperfusion injury in kidney transplantation: focus on ferroptosis, mitophagy and new antioxidants. *Antioxid. (Basel)*. **11**10.3390/antiox11040769 (2022). 10.3390/antiox11040769 PMC9024672 35453454

[18] Yang, Y. Y., Gong, D. J., Zhang, J. J., Liu, X. H. & Wang, L. Diabetes aggravates renal ischemia-reperfusion injury by repressing mitochondrial function and PINK1/Parkin-mediated mitophagy. *Am. J. Physiol. Ren. Physiol.* **317**, F852–f864. 10.1152/ajprenal.00181.2019 (2019). 10.1152/ajprenal.00181.2019 31390235

[19] Zhang, Z. et al. Unilateral partial nephrectomy with warm ischemia results in acute hypoxia inducible factor 1-Alpha (HIF-1α) and Toll-Like receptor 4 (TLR4) overexpression in a Porcine model. *PLoS One*. **11**, e0154708. 10.1371/journal.pone.0154708 (2016). 27149666 10.1371/journal.pone.0154708 PMC4858142

[20] Liu, H., Li, Y. & Xiong, J. The role of Hypoxia-Inducible Factor-1 alpha in renal disease. *Molecules* **27**10.3390/molecules27217318 (2022). 10.3390/molecules27217318 PMC9657345 36364144

[21] Fu, Z. J. et al. HIF-1α-BNIP3-mediated mitophagy in tubular cells protects against renal ischemia/reperfusion injury. *Redox Biol.* **36**, 101671. 10.1016/j.redox.2020.101671 (2020). 32829253 10.1016/j.redox.2020.101671 PMC7452120

[22] López-Cano, C. et al. Effect of type 2 diabetes mellitus on the Hypoxia-Inducible factor 1-Alpha expression. Is there a relationship with the clock genes?? *J. Clin. Med.* **9**10.3390/jcm9082632 (2020). 10.3390/jcm9082632 PMC7465909 32823749

[23] Choudhry, H. & Harris, A. L. Advances in Hypoxia-Inducible factor biology. *Cell. Metab.* **27**, 281–298. 10.1016/j.cmet.2017.10.005 (2018). 29129785 10.1016/j.cmet.2017.10.005

[24] Peek, C. B. et al. Circadian clock interaction with HIF1α mediates oxygenic metabolism and anaerobic Glycolysis in skeletal muscle. *Cell. Metab.* **25**, 86–92. 10.1016/j.cmet.2016.09.010 (2017). 27773696 10.1016/j.cmet.2016.09.010 PMC5226863

[25] Harreiter, J. & Roden, M. [Diabetes mellitus-Definition, classification, diagnosis, screening and prevention (Update 2019)]. *Wien Klin. Wochenschr*. **131**, 6–15. 10.1007/s00508-019-1450-4 (2019). 30980151 10.1007/s00508-019-1450-4

[26] Anders, H. J., Huber, T. B., Isermann, B. & Schiffer, M. CKD in diabetes: diabetic kidney disease versus nondiabetic kidney disease. *Nat. Rev. Nephrol.* **14**, 361–377. 10.1038/s41581-018-0001-y (2018). 29654297 10.1038/s41581-018-0001-y

[27] Ralto, K. M., Rhee, E. P. & Parikh, S. M. NAD(+) homeostasis in renal health and disease. *Nat. Rev. Nephrol.* **16**, 99–111. 10.1038/s41581-019-0216-6 (2020). 31673160 10.1038/s41581-019-0216-6 PMC7223841

[28] Giorgi, C. et al. Mitochondria and reactive oxygen species in aging and Age-Related diseases. *Int. Rev. Cell. Mol. Biol.* **340**, 209–344. 10.1016/bs.ircmb.2018.05.006 (2018). 30072092 10.1016/bs.ircmb.2018.05.006 PMC8127332

[29] Tang, C., He, L., Liu, J., Dong, Z. & Mitophagy Basic mechanism and potential role in kidney diseases. *Kidney Dis. (Basel)*. **1**, 71–79. 10.1159/000381510 (2015). 27536667 10.1159/000381510 PMC4934814

[30] Tang, C. et al. PINK1-PRKN/PARK2 pathway of mitophagy is activated to protect against renal ischemia-reperfusion injury. *Autophagy* **14**, 880–897. 10.1080/15548627.2017.1405880 (2018). 29172924 10.1080/15548627.2017.1405880 PMC6070003

[31] Li, N., Wang, H., Jiang, C. & Zhang, M. Renal ischemia/reperfusion-induced mitophagy protects against renal dysfunction via Drp1-dependent-pathway. *Exp. Cell. Res.* **369**, 27–33. 10.1016/j.yexcr.2018.04.025 (2018). 29704468 10.1016/j.yexcr.2018.04.025

[32] Jin, Q. et al. DUSP1 alleviates cardiac ischemia/reperfusion injury by suppressing the Mff-required mitochondrial fission and Bnip3-related mitophagy via the JNK pathways. *Redox Biol.* **14**, 576–587. 10.1016/j.redox.2017.11.004 (2018). 29149759 10.1016/j.redox.2017.11.004 PMC5691221

[33] Webster, K. A., Graham, R. M. & Bishopric, N. H. BNip3 and signal-specific programmed death in the heart. *J. Mol. Cell. Cardiol.* **38**, 35–45. 10.1016/j.yjmcc.2004.11.007 (2005). 15623420 10.1016/j.yjmcc.2004.11.007

[34] Yuan, C., Pu, L., He, Z. & Wang, J. BNIP3/Bcl-2-mediated apoptosis induced by Cyclic tensile stretch in human cartilage endplate-derived stem cells. *Exp. Ther. Med.* **15**, 235–241. 10.3892/etm.2017.5372 (2018). 29375685 10.3892/etm.2017.5372 PMC5763692

[35] Park, C. W. et al. BNIP3 is degraded by ULK1-dependent autophagy via MTORC1 and AMPK. *Autophagy* 9, 345–360, (2013). 10.4161/auto.23072 10.4161/auto.23072 PMC3590255 23291726

[36] Hanna, R. A. et al. Microtubule-associated protein 1 light chain 3 (LC3) interacts with Bnip3 protein to selectively remove Endoplasmic reticulum and mitochondria via autophagy. *J. Biol. Chem.* **287**, 19094–19104. 10.1074/jbc.M111.322933 (2012). 22505714 10.1074/jbc.M111.322933 PMC3365942

[37] Ishihara, M. et al. Sestrin-2 and BNIP3 regulate autophagy and mitophagy in renal tubular cells in acute kidney injury. *Am. J. Physiol. Ren. Physiol.* **305**, F495–509. 10.1152/ajprenal.00642.2012 (2013). 10.1152/ajprenal.00642.2012 23698117

[38] Tang, C. et al. Activation of BNIP3-mediated mitophagy protects against renal ischemia-reperfusion injury. *Cell. Death Dis.* **10**, 677. 10.1038/s41419-019-1899-0 (2019). 31515472 10.1038/s41419-019-1899-0 PMC6742651

[39] Hong, Z. et al. The HIF-1/ BNIP3 pathway mediates mitophagy to inhibit the pyroptosis of fibroblast-like synoviocytes in rheumatoid arthritis. *Int. Immunopharmacol.* **127**, 111378. 10.1016/j.intimp.2023.111378 (2024). 38141408 10.1016/j.intimp.2023.111378

[40] Costello, H. M., Johnston, J. G., Juffre, A., Crislip, G. R. & Gumz, M. L. Circadian clocks of the kidney: function, mechanism, and regulation. *Physiol. Rev.* **102**, 1669–1701. 10.1152/physrev.00045.2021 (2022). 35575250 10.1152/physrev.00045.2021 PMC9273266

[41] Rabinovich-Nikitin, I. et al. Mitochondrial autophagy and cell survival is regulated by the circadian clock gene in cardiac myocytes during ischemic stress. *Autophagy* **17**, 3794–3812. 10.1080/15548627.2021.1938913 (2021). 34085589 10.1080/15548627.2021.1938913 PMC8632283

[42] Schmitt, K. et al. Circadian control of DRP1 activity regulates mitochondrial dynamics and bioenergetics. *Cell. Metab.* **27**, 657–666e655. 10.1016/j.cmet.2018.01.011 (2018). 29478834 10.1016/j.cmet.2018.01.011

[43] James, M. T. et al. A Meta-analysis of the association of estimated GFR, albuminuria, diabetes mellitus, and hypertension with acute kidney injury. *Am. J. Kidney Dis.* **66**, 602–612. 10.1053/j.ajkd.2015.02.338 (2015). 25975964 10.1053/j.ajkd.2015.02.338 PMC4594211

[44] Adamovich, Y., Ladeuix, B., Golik, M., Koeners, M. P. & Asher, G. Rhythmic oxygen levels reset circadian clocks through HIF1α. *Cell. Metab.* **25**, 93–101. 10.1016/j.cmet.2016.09.014 (2017). 27773695 10.1016/j.cmet.2016.09.014

[45] Ma, Z. et al. Deletion of clock gene Bmal1 impaired the chondrocyte function due to disruption of the HIF1α-VEGF signaling pathway. *Cell. Cycle*. **18**, 1473–1489. 10.1080/15384101.2019.1620572 (2019). 31107137 10.1080/15384101.2019.1620572 PMC6592248

[46] Ghorbel, M. T., Coulson, J. M. & Murphy, D. Cross-talk between hypoxic and circadian pathways: cooperative roles for hypoxia-inducible factor 1alpha and CLOCK in transcriptional activation of the vasopressin gene. *Mol. Cell. Neurosci.* **22**, 396–404. 10.1016/s1044-7431(02)00019-2 (2003). 12691740 10.1016/s1044-7431(02)00019-2

[47] Dandavate, V. et al. Hepatic BMAL1 and HIF1α regulate a time-dependent hypoxic response and prevent hepatopulmonary-like syndrome. *Cell Metab* 36, 2038–2053.e (2035). 10.1016/j.cmet.2024.07.003 (2024). 10.1016/j.cmet.2024.07.003 39106859

[48] Suyama, K. et al. Circadian factors BMAL1 and RORα control HIF-1α transcriptional activity in nucleus pulposus cells: implications in maintenance of intervertebral disc health. *Oncotarget* **7**, 23056–23071. 10.18632/oncotarget.8521 (2016). 27049729 10.18632/oncotarget.8521 PMC5029610

[49] Zou, Y. F. et al. MicroRNA-30c-5p ameliorates hypoxia-reoxygenation-induced tubular epithelial cell injury via HIF1α stabilization by targeting SOCS3. *Oncotarget* **8**, 92801–92814. 10.18632/oncotarget.21582 (2017). 29190957 10.18632/oncotarget.21582 PMC5696223

[50] Catrina, S. B. & Zheng, X. Hypoxia and hypoxia-inducible factors in diabetes and its complications. *Diabetologia* **64**, 709–716. 10.1007/s00125-021-05380-z (2021). 33496820 10.1007/s00125-021-05380-z PMC7940280

[51] García-Pastor, C., Benito-Martínez, S., Moreno-Manzano, V., Fernández-Martínez, A. B. & Lucio-Cazaña, F. J. Mechanism and consequences of the impaired Hif-1α response to hypoxia in human proximal tubular HK-2 cells exposed to high glucose. *Sci. Rep.* **9**, 15868. 10.1038/s41598-019-52310-6 (2019). 31676796 10.1038/s41598-019-52310-6 PMC6825166

[52] Koh, M. Y. et al. Molecular mechanisms for the activity of PX-478, an antitumor inhibitor of the hypoxia-inducible factor-1alpha. *Mol. Cancer Ther.* **7**, 90–100. 10.1158/1535-7163.Mct-07-0463 (2008). 18202012 10.1158/1535-7163.MCT-07-0463

[53] Percie du Sert. The ARRIVE guidelines 2.0: updated guidelines for reporting animal research. *PLoS Biol.* **18**, e3000410. 10.1371/journal.pbio.3000410 (2020). 32663219 10.1371/journal.pbio.3000410 PMC7360023

[54] Wei, Q. & Dong, Z. Mouse model of ischemic acute kidney injury: technical notes and tricks. *Am. J. Physiol. Ren. Physiol.* **303**, F1487–1494. 10.1152/ajprenal.00352.2012 (2012). 10.1152/ajprenal.00352.2012 PMC3532486 22993069

[55] Lam, J. et al. A universal approach to analyzing transmission electron microscopy with ImageJ. *Cells* **10**10.3390/cells10092177 (2021). 10.3390/cells10092177 PMC8465115 34571826

