# Supplementary material for: Circadian gene BMAL1 ameliorates renal ischaemia-reperfusion injury in diabetic mice by enhancing mitophagy via the HIF-1/BNIP3 pathway
Source: Sci Rep. 2025 Jul 2;15:23001. doi: 10.1038/s41598-025-03515-5 (PMC12214787; doi:10.1038/s41598-025-03515-5)
Supplement: Supplementary file 1 — Supplementary Material 1 [file 41598_2025_3515_MOESM1_ESM.pdf]

Figure S1

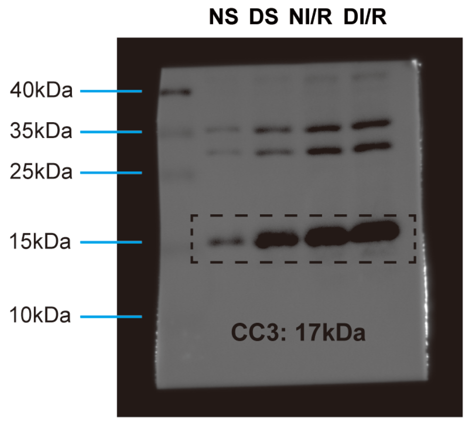

Original image of Figure 1G
with gray protein ladder.

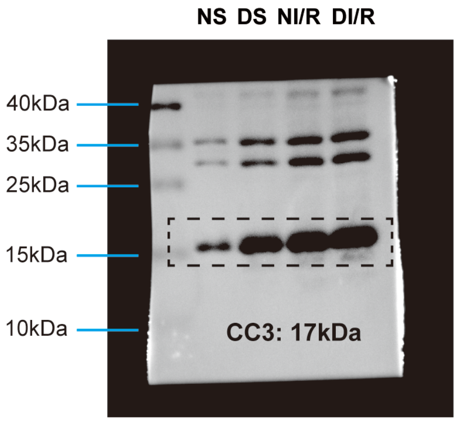

Original image of Figure 1G
with gray protein ladder.

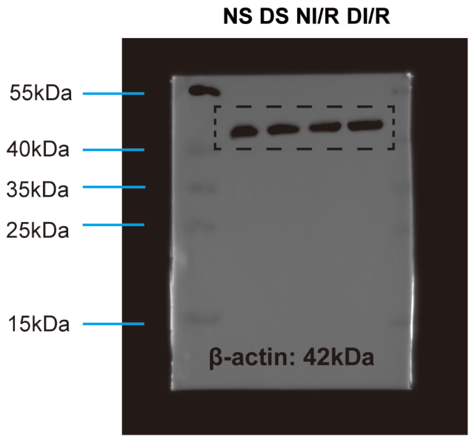

Original image of Figure 1G
with gray protein ladder.

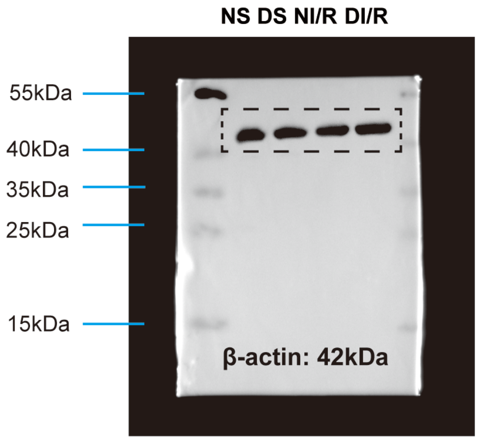

Original image of Figure 1G
with gray protein ladder.

Figure S2

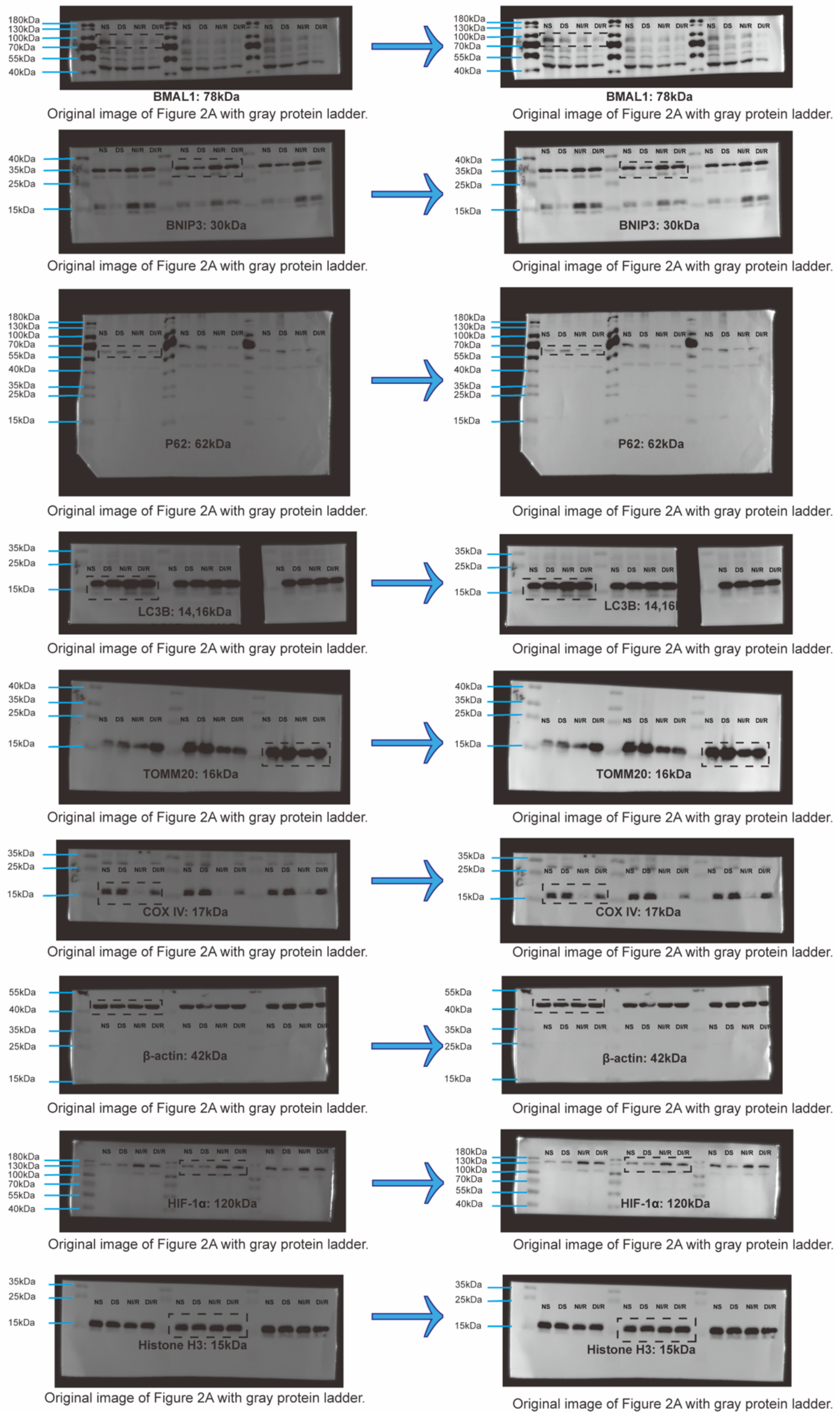
180kDa
130kDa
100kDa
70kDa
55kDa
40kDa
NS DS NI/R DI/R
BMAL1: 78kDa
Original image of Figure 2A with gray protein ladder.
BMAL1: 78kDa
Original image of Figure 2A with gray protein ladder.
40kDa
35kDa
25kDa
15kDa
BNIP3: 30kDa
Original image of Figure 2A with gray protein ladder.
BNIP3: 30kDa
Original image of Figure 2A with gray protein ladder.
180kDa
130kDa
100kDa
70kDa
55kDa
40kDa
35kDa
25kDa
15kDa
P62: 62kDa
Original image of Figure 2A with gray protein ladder.
P62: 62kDa
Original image of Figure 2A with gray protein ladder.
35kDa
25kDa
15kDa
LC3B: 14,16kDa
Original image of Figure 2A with gray protein ladder.
Original image of Figure 2A with gray protein ladder.
40kDa
35kDa
25kDa
15kDa
TOMM20: 16kDa
Original image of Figure 2A with gray protein ladder.
TOMM20: 16kDa
Original image of Figure 2A with gray protein ladder.
35kDa
25kDa
15kDa
COX IV: 17kDa
Original image of Figure 2A with gray protein ladder.
COX IV: 17kDa
Original image of Figure 2A with gray protein ladder.
55kDa
40kDa
35kDa
25kDa
15kDa
β-actin: 42kDa
Original image of Figure 2A with gray protein ladder.
β-actin: 42kDa
Original image of Figure 2A with gray protein ladder.
180kDa
130kDa
100kDa
70kDa
55kDa
40kDa
HIF-1α: 120kDa
Original image of Figure 2A with gray protein ladder.
HIF-1α: 120kDa
Original image of Figure 2A with gray protein ladder.
35kDa
25kDa
15kDa
Histone H3: 15kDa
Original image of Figure 2A with gray protein ladder.
Histone H3: 15kDa
Original image of Figure 2A with gray protein ladder.

Figure S3

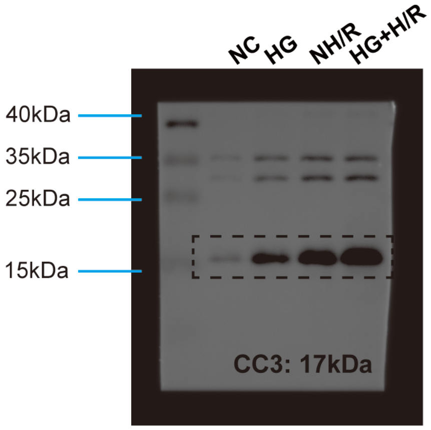

Original image of Figure 3E
with gray protein ladder.

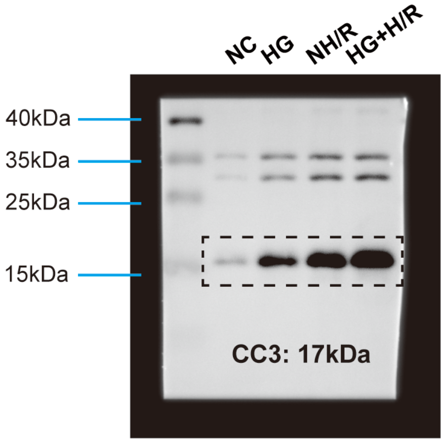

Original image of Figure 3E
with gray protein ladder.

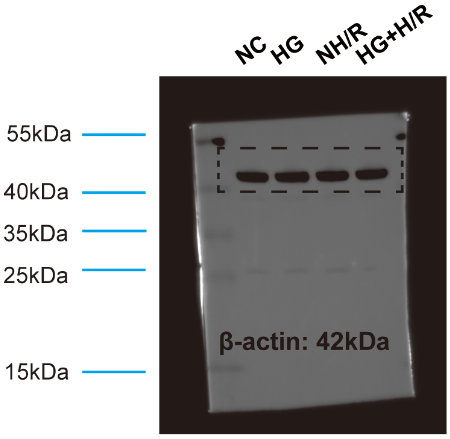

Original image of Figure 3E
with gray protein ladder.

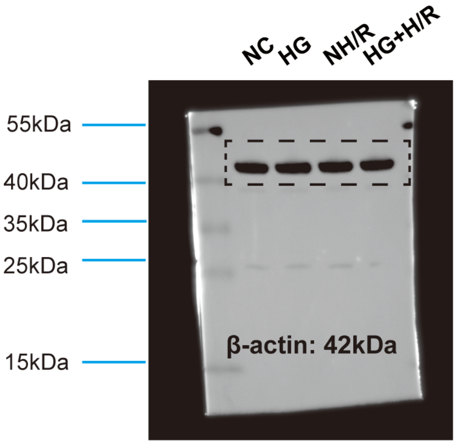

Original image of Figure 3E
with gray protein ladder.

Figure S4

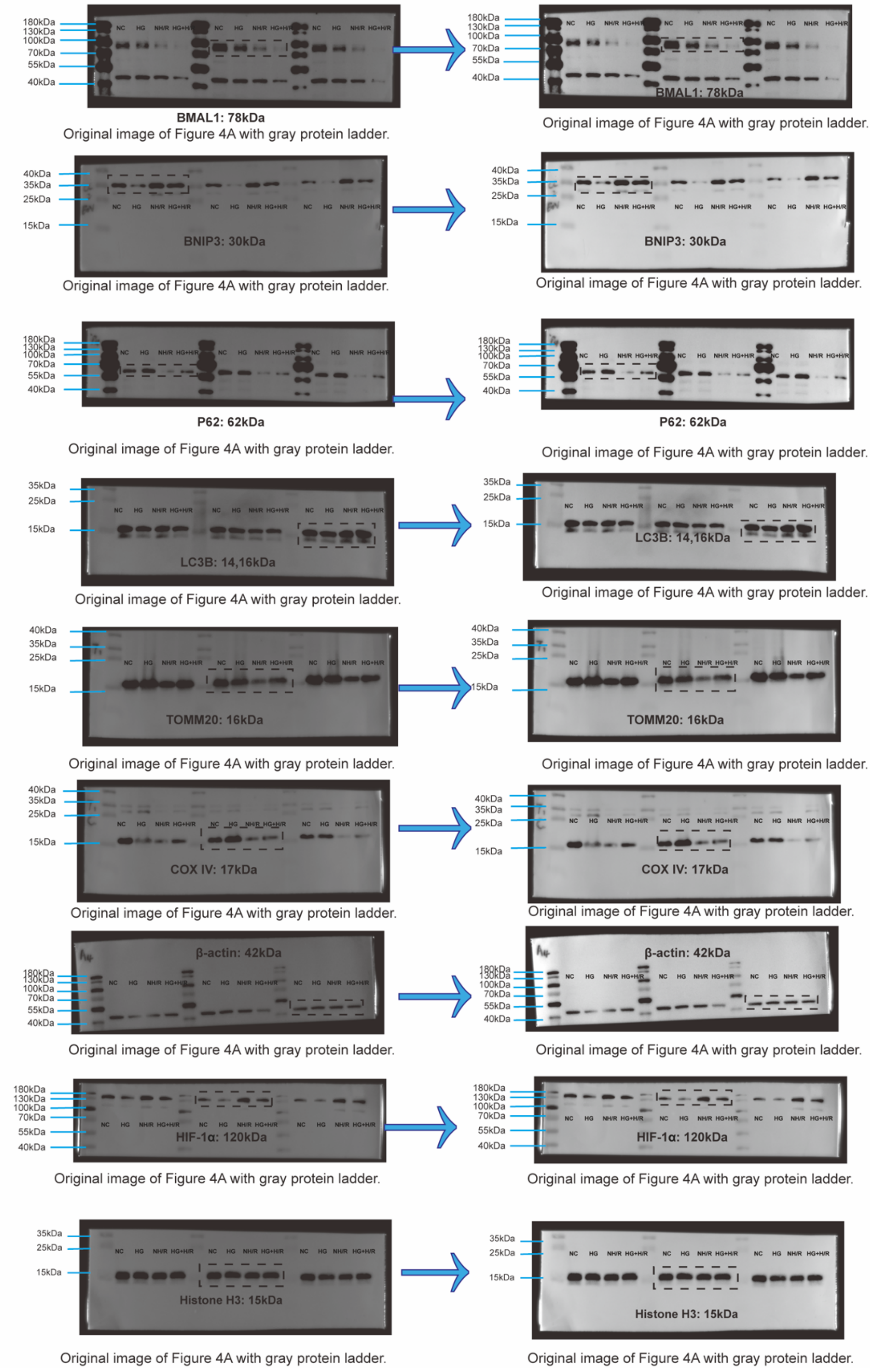

Original image of Figure 4A with gray protein ladder.

Figure S5

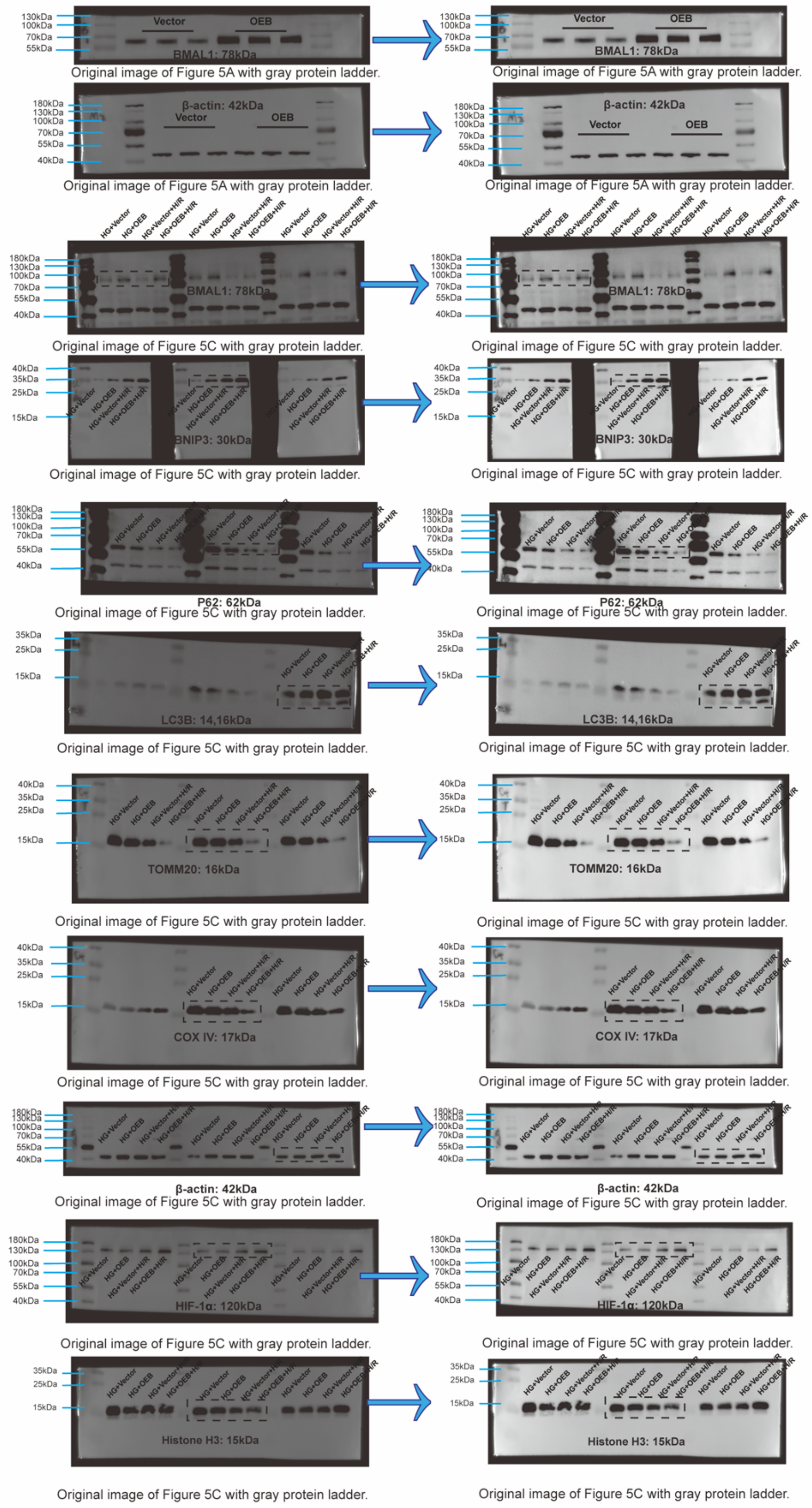
Vector
OEB
BMAL1: 78kDa
Original image of Figure 5A with gray protein ladder.
β-actin: 42kDa
Original image of Figure 5A with gray protein ladder.
HG+Vector
HG+OEB
HG+Vector+H/R
HG+OEB+H/R
BMAL1: 78kDa
Original image of Figure 5C with gray protein ladder.
BNIP3: 30kDa
Original image of Figure 5C with gray protein ladder.
P62: 62kDa
Original image of Figure 5C with gray protein ladder.
LC3B: 14,16kDa
Original image of Figure 5C with gray protein ladder.
TOMM20: 16kDa
Original image of Figure 5C with gray protein ladder.
COX IV: 17kDa
Original image of Figure 5C with gray protein ladder.
β-actin: 42kDa
Original image of Figure 5C with gray protein ladder.
HIF-1α: 120kDa
Original image of Figure 5C with gray protein ladder.
Histone H3: 15kDa
Original image of Figure 5C with gray protein ladder.
180kDa
130kDa
100kDa
70kDa
55kDa
40kDa
35kDa
25kDa
15kDa

Figure S6

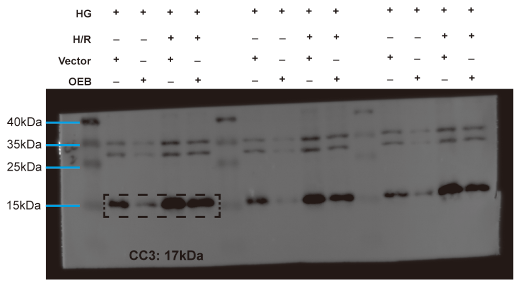

Original image of Figure 6E with gray protein ladder.

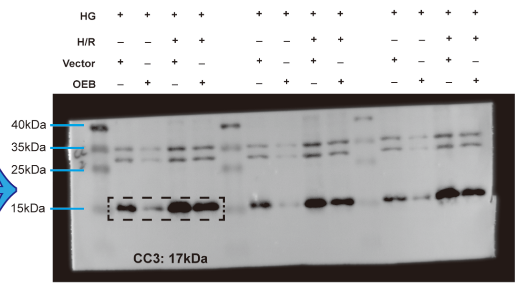

Original image of Figure 6E with gray protein ladder.

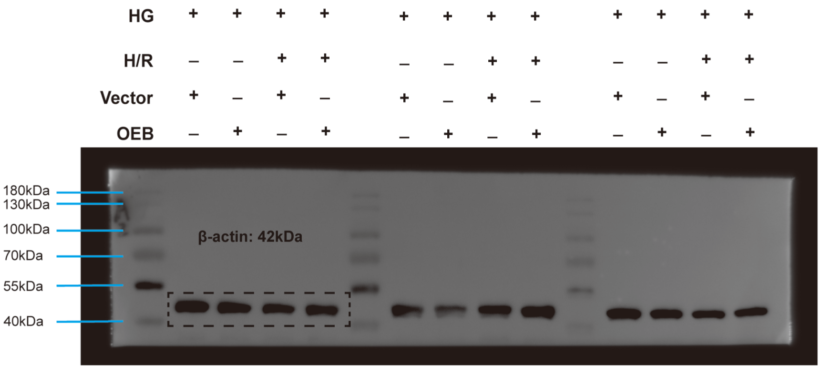

Original image of Figure 6E with gray protein ladder.

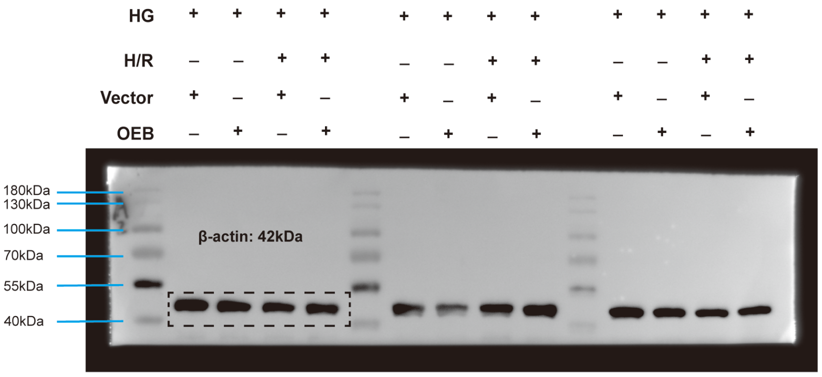

Original image of Figure 6E with gray protein ladder.

Figure S7

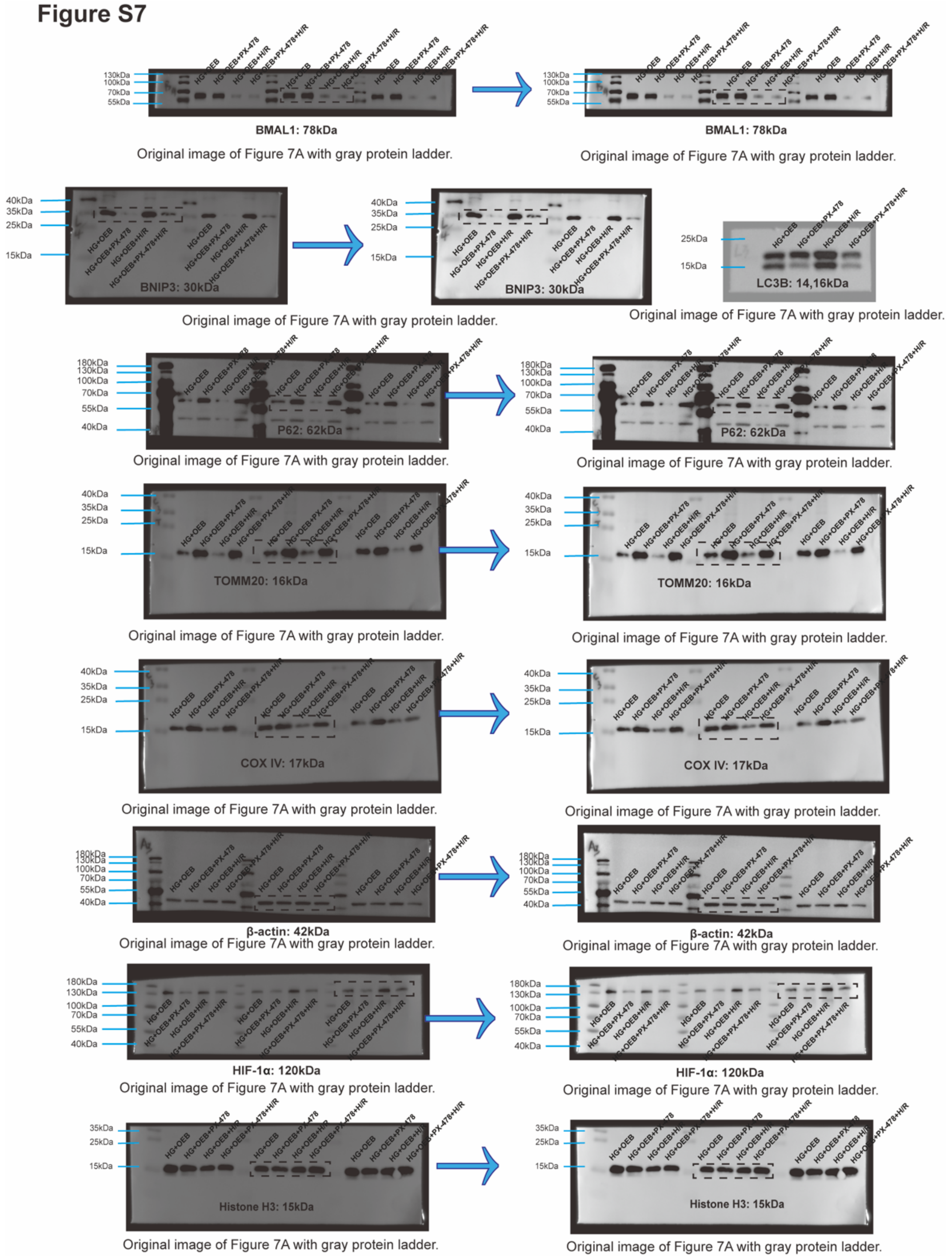

BMAL1: 78kDa
Original image of Figure 7A with gray protein ladder.
BMAL1: 78kDa
Original image of Figure 7A with gray protein ladder.
BNIP3: 30kDa
BNIP3: 30kDa
Original image of Figure 7A with gray protein ladder.
LC3B: 14,16kDa
Original image of Figure 7A with gray protein ladder.
P62: 62kDa
Original image of Figure 7A with gray protein ladder.
P62: 62kDa
Original image of Figure 7A with gray protein ladder.
TOMM20: 16kDa
Original image of Figure 7A with gray protein ladder.
TOMM20: 16kDa
Original image of Figure 7A with gray protein ladder.
COX IV: 17kDa
Original image of Figure 7A with gray protein ladder.
COX IV: 17kDa
Original image of Figure 7A with gray protein ladder.
β-actin: 42kDa
Original image of Figure 7A with gray protein ladder.
β-actin: 42kDa
Original image of Figure 7A with gray protein ladder.
HIF-1α: 120kDa
Original image of Figure 7A with gray protein ladder.
HIF-1α: 120kDa
Original image of Figure 7A with gray protein ladder.
Histone H3: 15kDa
Original image of Figure 7A with gray protein ladder.
Histone H3: 15kDa
Original image of Figure 7A with gray protein ladder.

Figure S8

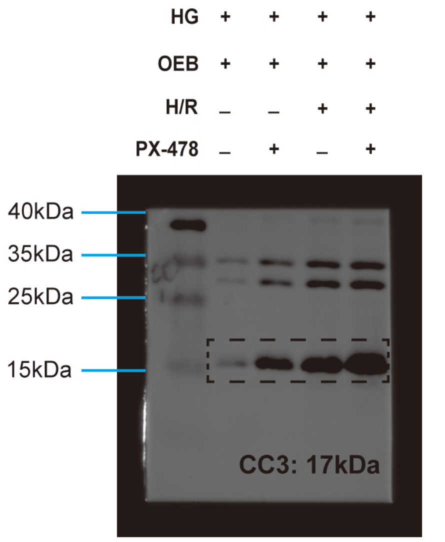

Original image of Figure 8E
with gray protein ladder.

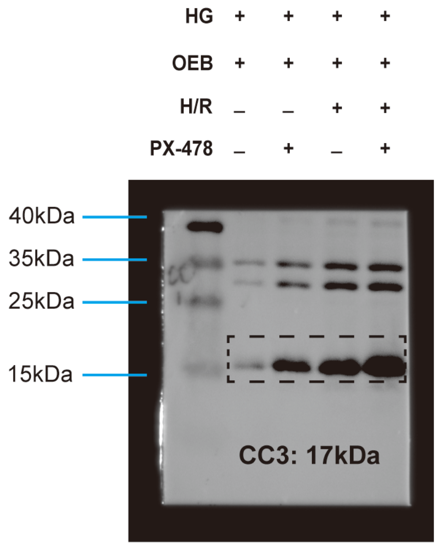

Original image of Figure 8E
with gray protein ladder.

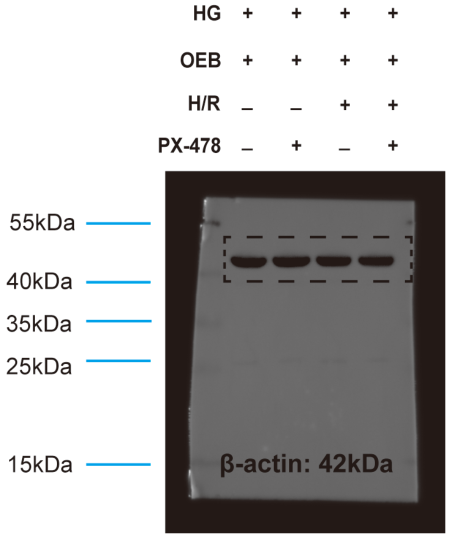

Original image of Figure 8E
with gray protein ladder.

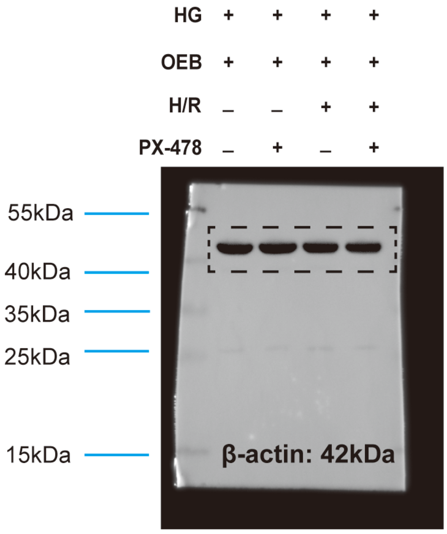

Original image of Figure 8E
with gray protein ladder.

Figure S9

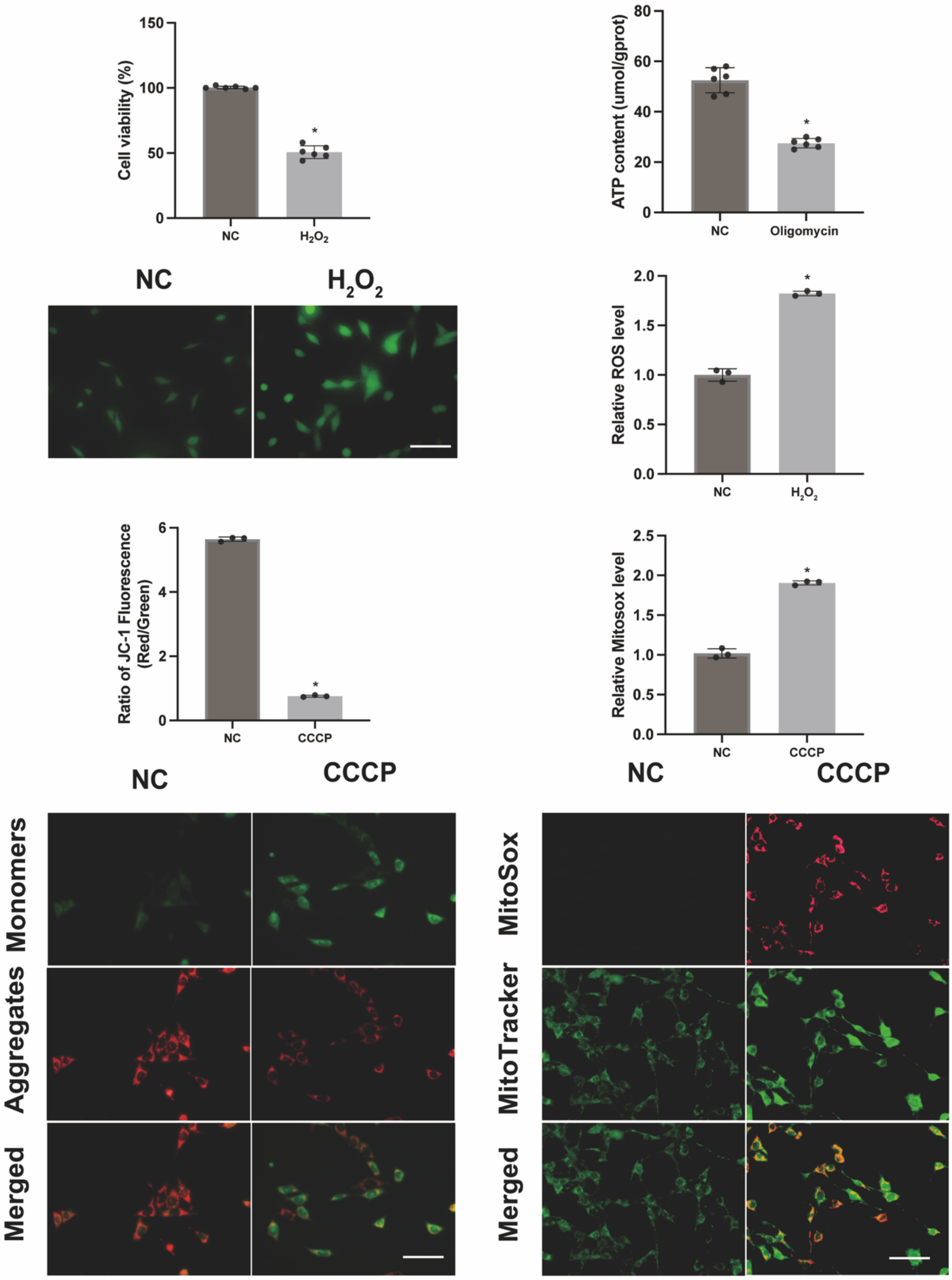
Cell viability (%)
150
100
50
0
NC
H2O2
ATP content (umol/gprot)
80
60
40
20
0
NC
Oligomycin
NC
H2O2
Relative ROS level
2.0
1.5
1.0
0.5
0.0
NC
H2O2
Ratio of JC-1 Fluorescence (Red/Green)
6
4
2
0
NC
CCCP
Relative Mitosox level
2.5
2.0
1.5
1.0
0.5
0.0
NC
CCCP
NC
CCCP
NC
CCCP
Monomers
Aggregates
Merged
MitoSox
MitoTracker
Merged
